# De-ubiquitinase USP35 promotes peritoneal dissemination of gastric cancer by regulating metabolic reprogramming

**DOI:** 10.1038/s41419-025-08322-4

**Published:** 2025-12-10

**Authors:** Lirong Yan, Moye Chen, LuLu Cai, Aoran Liu, Fang Li, Yuzhe Zhang, Xiaoli Peng, Yan Wang, RuiPeng Li, Jipeng Mei, Dan Zou, Xiaozhuo Gao, Yiwei Wang, Lina Wu, Ye Zhang

**Affiliations:** 1 The Laboratory of Cancer Institute, The First Hospital of China Medical University, Shenyang, 110001 China; 2 Department of Pharmacy, Personalized Drug Research and Therapy Key Laboratory of Sichuan Province, Sichuan Provincial People’s Hospital, School of Medicine, University of Electronic Science and Technology of China, Chengdu, 610072 China; 3 Department of Gastroenterology, The First Hospital of China Medical University, Shenyang City, Liaoning Province China; 4 Department of Oncology, Cancer Hospital of China Medical University, Liaoning Cancer Hospital and Institute, Shenyang, China; 5 Department of Pathology, Cancer Hospital of China Medical University, Liaoning Cancer Hospital and Institute, Shenyang, China; 6 Liaoning Province Key Laboratory for Phenomics of Human Ethnic Specificity and Critical Illness, Shenyang, Liaoning 110034 China; 7 Department of Molecular Morphology Laboratory, Shenyang Medical College, Shenyang, Liaoning 110034 China; 8 Department of Laboratory Medicine, Shengjing Hospital of China Medical University, No. 36, San Hao Street, Shenyang, Liaoning 110004 China; 9 Liaoning Clinical Research Center for Laboratory Medicine, Shenyang, China

**Keywords:** Cancer, Cancer metabolism

## Abstract

The enhanced adhesion between gastric cancer (GC) cells and peritoneal mesothelial cells (PMCs) is one of the key factors in the formation of the pre-peritoneal-metastasis adaptive microenvironment. USP35 belongs to the ubiquitin-specific protease family and is involved in regulating the occurrence and progression of various diseases. However, whether this gene can regulate the adhesion of GC cells to PMCs has not been clarified. The aim of this study was to identify the mechanism by which USP35 promotes the formation of the pre-peritoneal-metastasis adaptive microenvironment of GC and to find potential therapeutic targets. For the first time, we found that USP35 expression is upregulated in GC tissues, especially in peritoneal metastatic nodules, and is associated with poor prognosis. USP35 expression is the highest in MKN-45P, which is closely related to its high peritoneal metastasis potential. The mechanism of action involves several key steps. Firstly, the gene targets STING through de-ubiquitination and stabilizes its expression. Through this interaction, USP35/STING activates the HIF-1α/FAK pathway, promoting energy metabolism reprogramming and further improving the adhesion ability of GC cells. Secondly, exosome USP35 derived from GC cells has been shown to promote the mesothelial–mesenchymal transformation (MMT) of PMCs, preparing the “soil” for cancer cell adhesion and growth and contributing to the establishment of a pre-peritoneal-metastasis adaptive microenvironment. In summary, USP35 synergistically promotes the establishment of this environment through the dual mechanisms of regulating energy metabolic reprogramming of tumor cells and inducing the MMT of PMCs via the exosome pathway, providing a new theoretical basis for searching for therapeutic targets of gastric cancer with peritoneal dissemination.

## Introduction

The pre-peritoneal-metastasis adaptive microenvironment refers to a special environment that gradually forms in a local area of the peritoneum before tumor cells metastasize to it. This is due to the influence of some primary-tumor-related factors, and it is conducive to the subsequent colonization and metastasis of tumor cells. Regarding the occurrence of gastric cancer with peritoneal dissemination (GCPD), the tumor cells in the primary focus detach from this site and enter the abdominal cavity in the form of single free-floating cells. After activating the intracellular program to resist apoptotic signals in the abdominal cavity, the surviving tumor cells adhere to the single layer of peritoneal mesothelial cells (PMCs) attached to the peritoneal surface and then gradually enter the space beneath the peritoneum to form metastatic nodules [1]. In conclusion, the adhesion of gastric cancer (GC) cells to PMCs is a key factor in the establishment of the pre-peritoneal-metastasis adaptive microenvironment and may be important in the exploration of the mechanism of GCPD and the search for potential therapeutic targets of peritoneal cancer [2].

Energy metabolism reprogramming has been recognized as a universal feature of human cancers. Aerobic glycolysis, also known as the “Warburg effect”, is a typical metabolic phenotype of cancer cells. Even when there is sufficient oxygen to support oxidative phosphorylation, tumor cells still convert most glucose into lactate, ultimately leading to a large accumulation of lactate in the tumor microenvironment [3, 4]. Research shows that energy metabolism reprogramming can regulate the intercellular adhesion process [5]. However, whether it can regulate the adhesion of GC cells to PMCs has been rarely reported.

USP35 belongs to the ubiquitin-specific protease family and is involved in regulating the occurrence and progression of various diseases. Previous studies have reported that this gene can regulate ferroptosis in cancer cells by targeting ferroportin [6]. USP35 plays a crucial role in resisting endoplasmic reticulum stress-induced cell death by stabilizing RRBP1 [7]. When the mitochondrial membrane potential is normal, USP35 is mainly located in the mitochondria. However, during mitochondrial depolarization, it dissociates from the damaged mitochondria and translocates ectopically to the cytoplasm, which contributes to PARK2-mediated mitophagy [8]. This also suggests that USP35 may be involved in regulating the energy metabolism of GC cells and thus tumor progression, but whether it is involved in regulating the adhesion of tumor cells remains unclear.

Stimulator of interferon genes (STING) plays the role of a “double-edged sword” in tumor metastasis, and its final effect depends on the immune microenvironment, tumor characteristics, and activation mode. Studies have shown that Galectin-1 promotes tumor metastasis by mediating chronic activation of the STING pathway in tumors, driving the recruitment of myeloid derived suppressor cells [9]. However, glycolysis supports STING signaling to promote the anti-tumor function of dendritic cells [10]. STING can regulate anti-tumor immunity by stabilizing the expression of HIF-1α, increasing glycolysis and reducing oxidative phosphorylation in macrophages [11]. Nonetheless, whether this protein can affect the progression of GC by regulating metabolic reprogramming has not been revealed.

Exosomes can carry abundant proteins and RNAs, deliver these substances to target cells, and play an important role in signal transduction [12]. PMCs are the first line of defense against the attack of tumor cells on the peritoneum and are crucial in GCPD. Research shows that exosomes derived from tumor cells mediate peritoneal metastasis by cultivating the pre-metastatic niche, promoting MMT in PMCs, and inducing tumor growth and chemoresistance [13]. PMCs undergoing MMT lead to the exposure of the sub-mesothelial extracellular matrix, which promotes the adhesion and colonization of tumor cells and accelerates the occurrence of peritoneal metastasis [14]. Whether USP35 can disrupt the PMC barrier through the exosome pathway, thereby facilitating the attachment and growth of tumor cells, remains to be further explored.

In this study, we found that USP35 was significantly upregulated in GCPD, which is significantly associated with poor prognosis in GC patients. Subsequently, from two different perspectives, we comprehensively revealed the mechanism by which USP35 promotes the formation of the pre-metastatic adaptive microenvironment in GC. On one hand, this gene de-ubiquitinates STING, activates the HIF-1α/FAK signaling pathway, regulates energy metabolism reprogramming, and thus enhances the adhesion ability of GC cells. On the other hand, exosome USP35 derived from GC cells induces MMT in PMCs, creating a “soil” for the tumor metastasis cascade. This enables tumor cells with enhanced adhesion ability to survive and reproduce better once they reach the peritoneum. In conclusion, for the first time, this study reveals that abnormally expressed USP35 synergistically promotes the colonization of GCs in the peritoneum through a dual mechanism of regulating energy metabolism reprogramming in tumor cells and MMT in PMCs, forming a favorable pre-metastatic adaptive microenvironment for peritoneal metastasis. This provides a new theoretical basis for finding therapeutic targets for GCPD.

## Materials and methods

### Antibodies and reagents

An immunohistochemical kit was purchased from Fuzhou Maixin Biological Technology (KIT-9710, Fujian, China). Fetal Bovine Serum (FBS) was purchased from Vivacell (04-001-1ACS, Australia), and antibodies of FAK (sc-1688) and p-FAK (sc-81493) were obtained from Santa Cruz Biotechnology (Santa Cruz, CA, USA). Antibodies of HIF-1α (20960-1-AP), fibronectin (15613-1-AP), e-selectin (20894-1-AP), ICAM-1 (10831-1-AP), β-actin (66009-1-Ig), CD81 (66866-1-Ig), TSG101 (28283-1-AP), and calnexin (10427-2-AP), as well as MYC tag antibody (16286-1-AP), DYKDDDDK tag antibody (66008-4-1 g), HA tag antibody (51064-2-AP), HRP-conjugated Affinipure Goat Anti-Rabbit IgG(H + L) (SA00001-2), and HRP-conjugated Affinipure Goat Anti-Mouse IgG(H + L) (SA00001-1), were purchased from Proteintech (Wuhan, China). Antibodies of E-cadherin (3195S), α-SMA (19245S), ubiquitin (3933S), and STING (#13647) were obtained from Cell Signaling Technology (Danvers, MA, USA), while USP35 antibody was purchased from LifeSpan BioSciences (LS-C178984, USA). Rabbit (DA1E) mAb IgG XP® Isotype Control was purchased from Cell Signaling Technology (#3900, MA, USA), as was Mouse Anti-rabbit IgG (Conformation Specific) (L27A9) mAb (HRP Conjugate) (#5127, MA, USA). Cycloheximide (HY-12320), MG-132 (HY-13259), C-176 (HY-112906), 2-Deoxy-D-glucose (HY-13966), and PX-478 (HY-10231) were obtained from MedChemExpress, while PKH26 was purchased from Solarbio (D0030, China).

### Bioinformatic analysis

Gene Expression Profiling Interactive Analysis (GEPIA, http://gepia.cancer-pku.cn/), including RNA sequencing data from The Cancer Genome Atlas (TCGA) and GTEx databases, was used to detect differentially expressed USP35. Spearman’s correlation coefficient was used for correlation analysis, and differential genes were obtained by using the DESeq2 package. Gene Set Enrichment Analysis (GSEA) was carried out using the R packages “clustersProfiler”, “Enrichment”, and “ggplot2”. We collected GSE66229 and GSE62254 from the Gene Expression Omnibus (GEO, https://www.ncbi.nlm.nih.gov/gds/) to analyze USP35 expression in normal gastric tissue, non-PM GC, and GC with PM. In addition, the correlation of USP35 with overall survival was analyzed by using the Kaplan–Meier plotter in GSE62254. The survival package was used to test the proportional risk hypothesis and perform Cox regression analysis. All statistical analyses and visualizations were performed using R (version 4.3.2). Additionally, the UbiBrowser website (http://ubibrowser.bio-it.cn/ubibrowser_v3/) was used to predict USP35 de-ubiquitination targets.

### Immunohistochemistry assay

This study included 37 para-cancer and 102 gastric cancer tissues from the First Hospital of China Medical University, as well as peritoneal metastatic nodules corresponding to 13 of the patients involved. Inclusion criteria: histopathological diagnosis of gastric cancer; no other primary tumors; no prior preoperative antitumor therapies (neoadjuvant chemoradiotherapy, targeted therapy, immunotherapy, etc.); first tumor treatment limited to subtotal gastrectomy; and no severe dysfunction of the heart, lungs, liver, kidneys, or other major organs. Exclusion criteria: other concurrent malignant tumors; prior preoperative antitumor therapies (neoadjuvant chemoradiotherapy, targeted therapy, immunotherapy, etc.); history of tumor-related surgeries other than subtotal gastrectomy; and severe dysfunction of the heart, lungs, liver, kidneys, or other major organs. Clinicopathological parameters such as age, sex, tumor grade, tumor stage, and survival time were collected, and informed consent was provided by all patients. This study was approved by the Ethics Committee of the First Hospital of China Medical University (【2025】285).

Formalin-fixed tissues were paraffin-embedded and sectioned into 5 μm slices for hematoxylin and eosin (H&E) and immunohistochemical (IHC) staining. Deparaffinized sections were treated with 3% H₂O₂ at room temperature for 10 min to quench endogenous peroxidase activity, then blocked with goat serum at room temperature for 30 min. After overnight incubation with primary antibody at 4 °C, sections were incubated with secondary antibody (10 min) and biotin-labeled horseradish peroxidase (10 min) sequentially. Antibody binding was visualized using a 3,3’-diaminobenzidine tetrahydrochloride (DAB) kit (Maixin, China), and IHC staining was finally observed under an inverted phase-contrast microscope. Staining intensity was evaluated using a 4-point scale, where a score of 0 indicated no staining, 1 represented low staining, 2 denoted intermediate staining, and 3 corresponded to high staining. For the staining area, a 5-tier scoring system was applied: ≤5% of the area was scored 0, 5–25% was scored 1, 26–50% was scored 2, 51–75% was scored 3, and >75% was scored 4. Multiply the staining intensity by the staining area to determine the final classification result: a total score of 0 was defined as negative (−); 1–4 points as weakly positive (+); 5–8 points as positive (++); and 9–12 points as strongly positive (+++). All final scores were independently assigned by three pathologists to ensure objectivity.

### Cell culture

In our previous study, the high-potential peritoneal dissemination cell line MKN-45P was induced by its parent, MKN-45, purchased from the Academy of Military Medical Science (Beijing, China) [15]. GES-1, AGS, and HGC-27 were obtained from the same institution. Peritoneal mesothelial HMrSV5 cells were purchased from ORiCells Biotechnology (Shanghai, China). All cell lines have STR identification certificates and were also subjected to mycoplasma testing. GES-1, AGS, HGC-27, MKN-45P, and MKN-45 were cultured in RPMI1640 (Vivacell, USA), and HMrSV5 was cultured in DMEM (Gibco, USA) containing 10% FBS at 37 °C in an atmosphere of 5% CO2.

### Lentivirus and plasmid transfection

The lentivirus for USP35 knockdown, the plasmid for USP35 overexpression and mutant forms, and ubiquitin plasmid were meticulously produced and kindly provided by Sangon Biotech (Shanghai, China). Meanwhile, the lentivirus for STING overexpression was supplied by Shanghai GeneChem Co., Ltd. JetPRIME® (101000046, Polyplus, France), a reliable transfection reagent, was employed to introduce the plasmids into cells following the manufacturer’s detailed protocol. To facilitate the transfection of cells with the USP35 knockdown lentivirus and the STING overexpression lentivirus, polybrene was utilized. As controls, cells intended for plasmid and lentivirus transfection were treated with an empty vector, ensuring the accuracy and reliability of subsequent experimental results.

### RNA sequencing

USP35-overexpressing MKN-45 cells and their empty vector control counterparts were sequenced on the Illumina HiSeq 2500 platform (conducted by Novo-gene Co., Ltd., China) to generate mRNA expression profiles. Total RNA samples were collected via Trizol lysis 24 h post-transfection. Nanodrop checked A260/A280 (1.8–2.1) and A260/A230 ( ≥ 2.0), and Agilent 2100 ensured RNA quality (RIN ≥ 8.0). We used 1 μg of qualified RNA to enrich mRNA with oligo (dT) beads. After fragmentation, mRNA was reverse-transcribed to ds cDNA. Libraries were built via end repair, adapter ligation, AMPure XP screening, and PCR, with QC confirming concentration ≥10 nmol/L and a main peak of 250–350 bp. PE150 sequencing was performed on Illumina HiSeq 2500 (Q30 ≥ 85%), and raw data were filtered to Clean Reads aligned to human GRCh38 for quantification.

DEGs were screened using DESeq2 (|log₂FC | > 1, padj<0.05). To sustain proliferation and adhesion, tumor cells dynamically reprogram energy metabolism and may favor either glycolysis or mitochondrial-related metabolic processes (including oxidative phosphorylation [OXPHOS], tricarboxylic acid cycling [TCA], fatty acid oxidation [FAO]). Thus, we developed a cellular energy metabolism bias score: SCORE = z_Gly(s) − [z_OXPHOS(s) + z_TCA(s) + z_FAO(s)]/3. Here, z_Gly(s), z_OXPHOS(s), z_TCA(s), and z_FAO(s) are all pathway-specific normalized means. Calculation: For each pathway (P) and sample (s), first compute the mean normalized expression of genes within P for s (x_P(s)); then, standardize across samples to obtain z_P(s) = (x_P(s) - μ_P)/σ_P (μ_P and σ_P = the total mean and standard deviation of pathway P). A higher SCORE indicates a stronger glycolytic tendency; a lower SCORE suggests a bias toward mitochondrial processes. The Welch t-test was used to compare the statistical differences between the scores of the two groups.

### RNA isolation and qRT-PCR

Total RNA was isolated using TRNzol Universal (DP424, TIANGEN, China). The absorbance at 260 nm was measured to quantify the concentration of total RNA. Reverse transcription was carried out with the PrimeScript™ RT Reagent Kit (RR047A, Takara, Japan). For quantitative real-time PCR (qRT-PCR), the SYBR Premix Ex TaqII (RR820A, Takara, Japan) was utilized. The measurements were normalized against β-actin, and the relative expression was calculated using the 2^−ΔΔCt^ method. The primer sequences are presented in Table S1.

### Western blotting

Proteins were extracted from the treated cells and quantified. Equal amounts of total protein were electrophoretically separated using the Mini-PROTEAN Tetra Cell system (Bio-Rad Laboratories Inc. Gels) and transferred onto a polyvinylidene fluoride (PVDF) membrane. This membrane was incubated with Protein Free Rapid Blocking Buffer (PS108P, Epizyme, China) at room temperature for 30 min and then incubated with the primary antibody at 4 °C overnight. On the next day, it was incubated with the secondary antibody at room temperature for 1 h. Finally, the membrane was visualized using BeyoECL Plus (P0018S, Beyotime, China).

### Metabolism assay

An ATP Assay Kit (S0027, Beyotime, China), glucose kit (A154-1-1, China) and lactic acid assay kit (A019-1-1, China) were used to detect the levels of ATP, glucose absorption, and lactic acid production in gastric cancer cells. All steps followed the operating instructions for the reagents.

### Adhesion assay

HMrSV5 cells were inoculated into 24-well plates and cultured at the bottoms to form single layers. MKN-45 and MKN-45P cells were stained in a 37°C incubator with DiD cell labeling solution (V22887, Invitrogen™) for 20 min and inoculated with HMrSV5 at a density of 1 × 10^5^ cells per well. After co-incubation for 6 h, the cells were photographed under a microscope with white light and a fluorescent field of view.

### Co-immunoprecipitation assay

When the cells reached 90% confluence, the culture medium was aspirated, and the cells were washed once with pre-cooled PBS. The residual liquid was completely aspirated. After collecting the cells, the experiment was carried out according to the operating instructions of the co-immunoprecipitation kit (abs955, absin, China). Finally, the samples were analyzed via Western blotting.

### Exosome extraction and identification

After the treated MKN-45 and MKN-45P cells reached 70–80% confluence, the culture medium was replaced with one containing exosome-depleted FBS. After 48 h, the conditioned medium (CM) was collected, centrifuged at 1000 rpm for 5 min, and then filtered through a 0.22 μm filter (SLGP033NS, Millipore). Exosomes were extracted according to the operating instructions of the exoEasy Maxi Kit [16] (76064, QIAGEN, Germany), and their morphological characteristics were observed via electron microscopy. The exosomes were resuspended in PBS containing 1% glutaraldehyde. Then, 20 μl of each sample was dropped onto a carbon-coated electron microscopy grid and left to stand at room temperature for 1 min. The exosome samples were stained with 20 μl of 2% phosphotungstic acid for 2 min, and their morphological characteristics were observed and photographed using a JEM-1200EX electron microscope. According to the manufacturer’s instructions, the absolute diameter size distribution of the isolated exosomes was analyzed using a NanoSight NS300 instrument (Malvern Instruments, Malvern, UK).

### Label-free proteomics

Exosomes derived from GES-1, MKN-45, and MKN-45P cells were collected for protein quantification. Label-free quantification is a method that analyzes proteolytic peptides via mass spectrometry without the need for expensive isotope labels as internal standards. In this analysis, we used version 2.2 of the Proteome Discoverer software. This technology was implemented using ECHO BIOTECH (Beijing, China).

### Transwell assay

Cells in the logarithmic growth phase were seeded into the upper chamber of a transwell (Corning, New York, USA) at a density of 5 × 10⁴ HMrSV5 cells per well and cultured for 24 h. After fixing the membrane with methanol for 20 min, the cells were stained with crystal violet. Photographs were taken using an optical microscope, and the cells were counted and analyzed using ImageJ.

### Peritoneal dissemination model of gastric cancer

Twenty-four female BALB/c nude mice aged 4-6 weeks were obtained from Huafukang Experimental Animal Technology Co., LTD. (Beijing, China), and housed in a specific pathogen-free (SPF) environment. Considering that MKN-45P cells have high potential for peritoneal dissemination, they were selected to establish a mouse model of GCPD. After one week of acclimation, the 24 mice were randomly divided into 4 groups (6 mice in each group): the control group, the USP35 knockdown group, the STING overexpression group, and the USP35 knockdown combined with STING overexpression group. Each mouse was intraperitoneally injected with approximately 5 × 10⁶ cells suspended in 200 μl of PBS and sacrificed two weeks after the injection.

Twenty-four female BALB/c nude mice aged 4-6 weeks were obtained from Huafukang Experimental Animal Technology Co., LTD. (Beijing, China). Specifically, the nude mice were intraperitoneally injected with exosomes derived from MKN-45P cells with stable knockdown of USP35, MKN-45 cells with overexpression of USP35 (100 μg/200 μl PBS), or control exosomes on Day 1, Day 3, and Day 5. On Day 5, 5 × 10⁶ MKN-45 and MKN-45P cells in 200 μl PBS were simultaneously injected intraperitoneally. All nude mice were sacrificed by cervical dislocation on Day 19.

No blinding method was employed in this animal study. The experimenters were aware of the group assignments during the operation and recording processes. Before sacrificing the mice, ^18^FDG was injected into the tail vein. After waiting for 30 min, a micro-PET scan was performed. Metis Console software was used for subsequent scanning and analysis. Peritoneal metastatic tumors and the peritoneum of the mice were collected for H&E staining and IHC detection. The IHC scoring criteria are consistent with the aforementioned immunohistochemical staining of human GC tissues. All animal experiments were conducted in accordance with the protocol approved by the Welfare and Ethics Committee of China Medical University (CMU20241780).

### Statistical analysis

The results were analyzed using SPSS 26.0 (SPSS, Chicago, IL) and GraphPad Prism 8.0 (GraphPad Software, LLC, San Diego, USA). All experiments were independently repeated at least three times, and the data were presented as mean ± SD. The Levene test or Bartlett test was used to assess the homogeneity of variances among the statistical comparison groups. When comparing between two groups, if the data followed a normal distribution, Student’s two-tailed t-test was used; if the data were skewed, a non-parametric test was employed. When making comparisons among multiple groups, one-way analysis of variance (ANOVA) was used. The chi-square test was employed to evaluate the relationship between USP35 expression and clinicopathological parameters of GC patients, and *P* < 0.05 was considered statistically significant.

## Results

### USP35 is associated with poor prognosis in GC

**Fig. 1:**
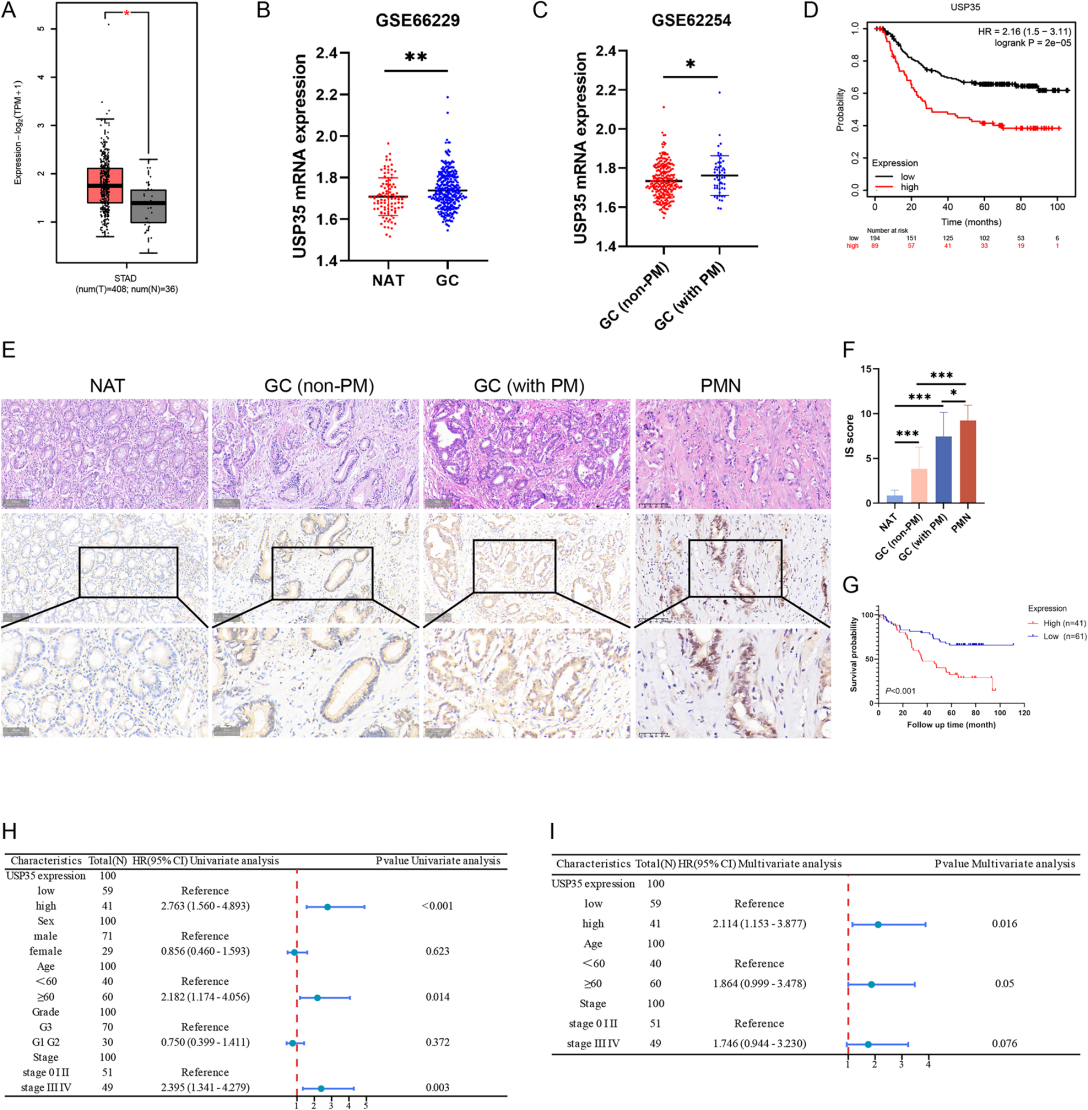
USP35 expression characteristics in GC. **A, B** The GEPIA and GSE66229 databases were used to analyze the differential expression of USP35 in normal and GC tissues. **C** Differential expression of USP35 in non-PM GC and GC with PM was analyzed in GSE62254. **D** Kaplan–Meier survival analysis demonstrated the association of USP35 expression with prognosis in GC in GSE62254. **E, F** Immunohistochemical staining showing USP35 expression in GC progression; scale bars: 50μm and 100μm. **G** Kaplan–Meier survival analysis of the association of USP35 expression with OS. **H, I** Univariate and multivariate COX regression analysis of patients with GC. Data are presented as the mean ± SD. Gene Expression Profiling Interactive Analysis, GEPIA; gastric cancer, GC; non-peritoneal metastasis, non-PM; peritoneal metastasis, PM; peritoneal metastatic nodule, PMN; overall survival, OS. *P < 0.05; **P < 0.01; ***P < 0.001.

The TCGA and GSE66229 datasets showed that USP35 was upregulated in GC tissues compared with normal tissues (Fig. 1A, B). The latter dataset indicated that this gene was also upregulated in GC tissues with peritoneal metastasis compared with those without it (Fig. 1C). Survival analysis of GSE62254 using the Kaplan–Meier method showed that high expression of USP35 was associated with poor prognosis in GC patients (Fig. 1D). Subsequently, IHC staining confirmed that USP35 expression gradually increased during GC progression, from normal adjacent tissue through to GC tissue without peritoneal metastasis, GC tissue with peritoneal metastasis, and peritoneal metastatic nodules (Fig. 1E, F). Analysis of clinicopathological parameters suggested that USP35 expression was significantly correlated with the stage and peritoneal metastasis of GC patients (Table 1). Kaplan–Meier survival analysis also showed that patients with high USP35 expression had a poor prognosis (Fig. 1G). Univariate and multivariate COX regression analyses revealed that high expression of USP35 was an independent risk factor affecting the prognosis of the patients (Fig. 1H–I).

**Table 1:** The association of USP35 expression with clinicopathological parameters in gastric cancer.

Variables	*N*	USP35 expression			
		High n (%)	Low n (%)	Chi-square value	*P* value
**Age**				1.044	0.307
≥60	61	27 (44.3)	34 (55.7)		
<60	41	14 (34.1)	27 (65.9)		
**Sex**				0.220	0.639
Male	72	30 (41.7)	42 (58.3)		
Female	30	11 (36.7)	19 (63.3)		
**T stage**				8.921	**0.003**
T1-2	32	6 (18.8)	26 (81.2)		
T3-4	70	35 (50.0)	35 (50.0)		
**N stage**				6.892	**0.009**
N0	51	14 (27.5)	37 (72.5)		
N1-3	51	27 (52.9)	24 (47.1)		
**pStage**				7.774	**0.005**
Ⅰ+Ⅱ	52	14 (26.9)	38 (73.1)		
Ⅲ+Ⅳ	50	27 (54.0)	23 (46.0)		
**Grade**				1.699	0.192
G1 + G2	30	15 (50.0)	15 (50.0)		
G3	72	26 (36.1)	46 (63.9)		
**Peritoneal metastasis**				9.193	**0.002**
Yes	20	14 (70.0)	6 (30.0)		
No	82	27 (32.9)	55 (67.1)		

### USP35 enhances the adhesion of GC cells by regulating energy metabolism reprogramming

**Fig. 2:**
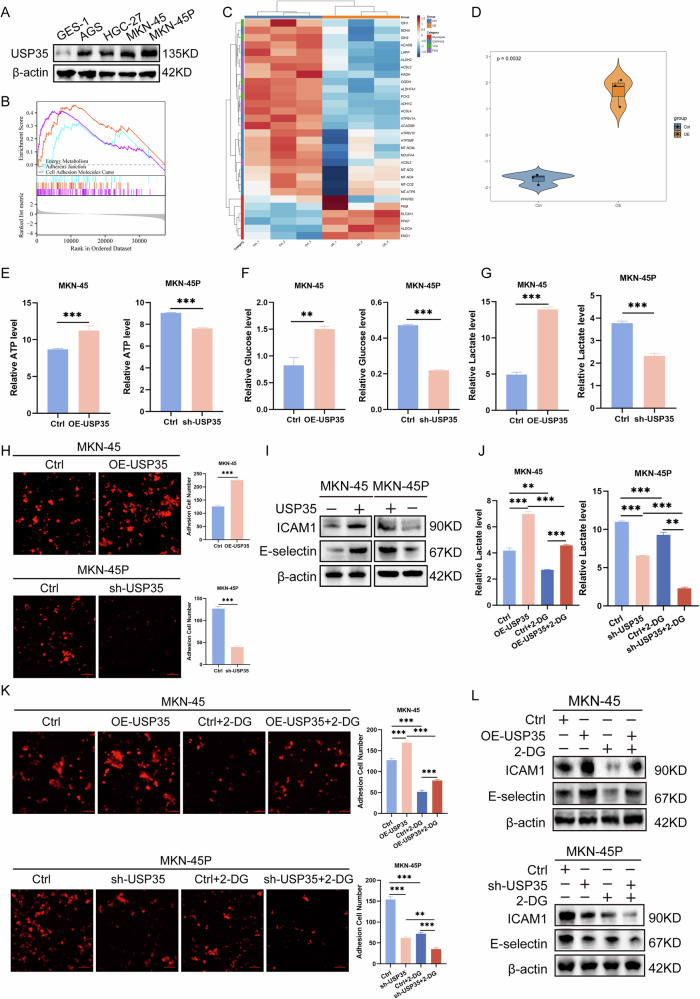
USP35 enhances the colonization ability of GC cells by regulating energy metabolism reprogramming. **A** Western blotting detected the background expression of USP35. **B** Differential genes were grouped according to the median expression of USP35 in the TCGA-STAD database, and GSEA was performed. **C** RNA-seq analysis assessed the expression changes in metabolism-related genes after USP35 overexpression. **D** Cellular energy metabolism bias score comparison between USP35-overexpressing and control MKN-45 cells. **E–G** The effects of USP35 on the metabolites in glycolysis (*n* = 3). **H, I** Adhesion assay and Western blotting disclosed the effect of USP35 on the colonization ability of GC cells (*n* = 5); scale bar: 500μm. **J** Lactate production in GC cells in the presence of 2-DG (*n* = 3). **K, L** Adhesion assay and Western blotting showed the effects of 2-DG on the colonization ability of GC cells (*n* = 5); scale bar: 500μm. Data are presented as the mean ± SD. The Cancer Genome Atlas Program, TCGA; stomach adenocarcinoma, STAD; 2-Deoxy-D-glucose, 2-DG. *P < 0.05; **P < 0.01; ***P < 0.001.

At the cellular level, we first used transwell and adhesion experiments to confirm that the high-peritoneal metastasis cell line MKN-45P induced by our team had stronger migration and adhesion ability than the parental gastric cancer cell line MKN-45 and was more prone to peritoneal metastasis (Fig. S1). Subsequently, Western blotting confirmed that compared to that in the normal gastric mucosal cell line GES-1, USP35 expression was higher in GC cell lines, and it was the highest in MKN-45P cells, indicating that USP35 is closely related to high peritoneal metastasis in MKN-45P cells (Fig. 2A). In order to explore the mechanism of USP35 promoting GC progression, we used the TCGA gastric cancer dataset for GSEA and found that USP35 was positively correlated with energy metabolism and cell adhesion (Fig. 2B). RNA sequencing results showed that compared with those in the control group, glycolysis-related genes were significantly upregulated in USP35-overexpressing group (Fig. 2C). Additionally, cellular energy metabolism bias scores revealed that this group had significantly higher SCORE values than the control group, suggesting USP35 influences GC progression primarily by regulating glycolysis (Fig. 2D). Subsequently, to determine the function of USP35 expression in GC cells, we overexpressed USP35 in AGS, HGC-27, and MKN-45 cells and knocked down USP35 in the induced high-potential peritoneal dissemination cell line MKN-45P (Fig. S2A–D). We found that USP35 expression could upregulate glucose uptake, lactate, and ATP levels in different GC cells (Fig. 2E–G, Fig. S3A–C). Adhesion assays showed that USP35 expression promoted the colonization ability of GC cells with HMrSV5 (Fig. 2H, Fig. S3D). Moreover, Western blot analysis indicated that USP35 knockdown could downregulate the expression of the adhesion molecules e-selectin and ICAM1, while overexpressing USP35 had the opposite effect, which further facilitated cell colonization (Fig. 2I, Fig. S3E).

To investigate whether energy metabolism reprogramming is related to the formation of the pre-metastatic adaptive microenvironment in the peritoneum, we treated the USP35-knockdown and USP35-overexpressing GC cells with 2-DG. The results showed that 2-DG significantly inhibited glycolysis in both types of cells (Fig. 2J). In addition, adhesion and Western blot assays showed that compared with that of the USP35-overexpressing group, the colonization ability of GC cells with HMrSV5 in the USP35-overexpressing combined with 2-DG group decreased, while the adhesion ability of the USP35 knockdown combined with 2-DG group decreased more significantly (Fig. 2K–L). These results indicate that USP35 promotes the colonization ability of GC cells by enhancing cellular glycolysis.

### USP35 stabilizes the expression of STING by mediating its de-ubiquitination

**Fig. 3:**
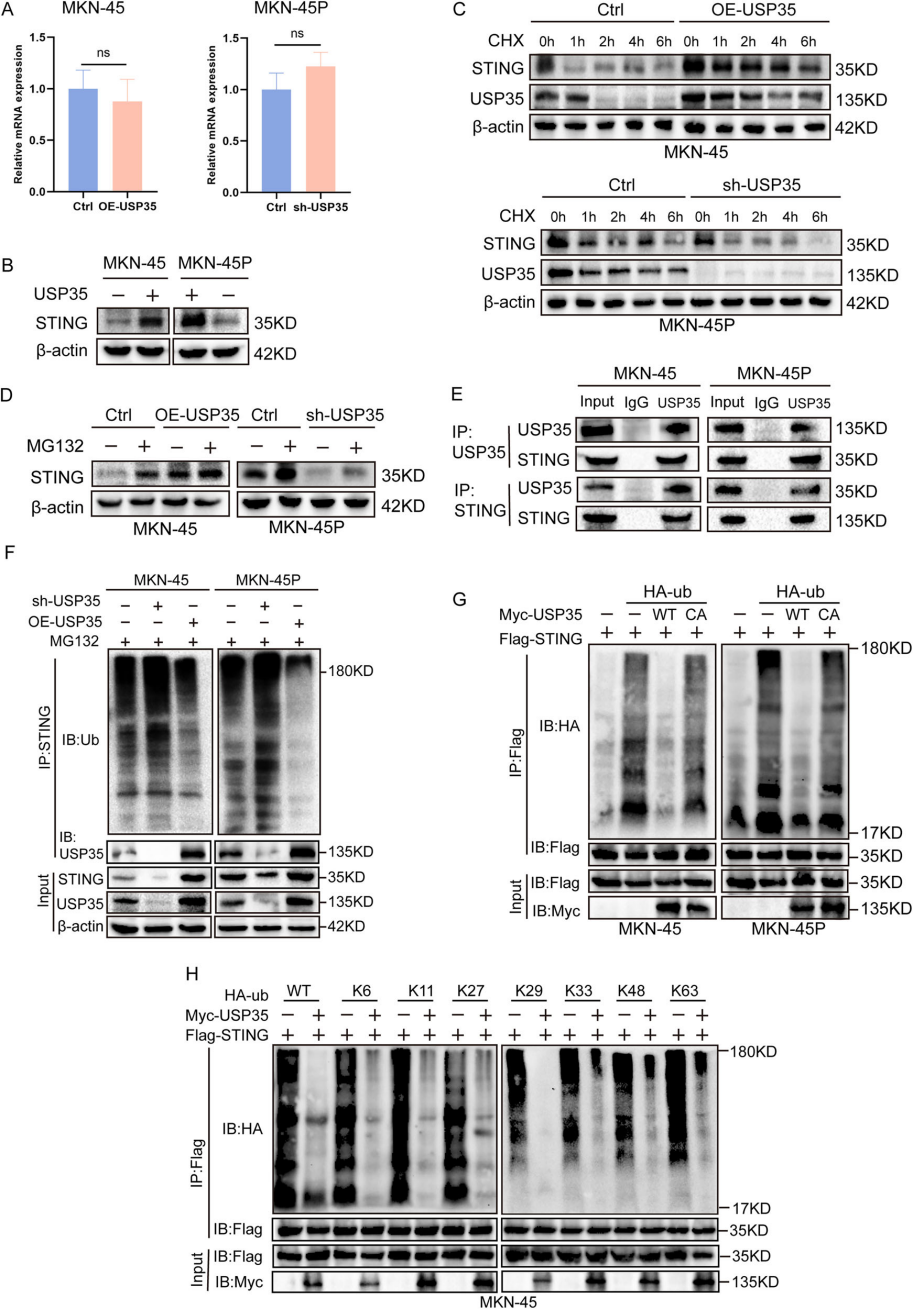
USP35 stabilizes STING expression by mediating de-ubiquitination. **A** RT-qPCR detected the effect of USP35 on STING expression at the mRNA level (*n* = 3). **B** The effect of USP35 on STING expression at the protein level. **C** The effect of USP35 on endogenous STING levels in GC cells treated with CHX (50 µg/mL). **D** STING expression in OE-USP35 and sh-USP35 GC cells. The cells were treated with MG132 (20 µM) for 6 h before harvest. **E** The interaction of USP35 and STING was detected via co-immunoprecipitation. **F** The effect of USP35 on the de-ubiquitination of STING. Prior to lysis, the cells were treated with MG132 for 6 h. **G** MKN-45 and MKN-45P cells were co-transfected with STING-Flag along with either Myc-USP35^WT^ or Myc-USP35^C450A^ and HA-ubiquitin for 24 h. Subsequently, cell lysates were subjected to immunoprecipitation, followed by a denaturation assay, to examine ubiquitination levels. **H** MKN-45 cells expressing the specified plasmids for 48 h were analyzed using Denature-IP using an anti-HA antibody, followed by immunoblotting with anti-Flag, anti-HA, or anti-Myc antibodies. Data are presented as the mean ± SD. no statistical significance, ns quantitative real-time PCR, RT-qPCR Cycloheximide, CHX overexpression, OE short hairpin, sh gastric cancer, GC.

As a de-ubiquitinase, USP35 mainly stabilizes the expression of downstream molecules through the de-ubiquitination pathway. Therefore, we used the UbiBrowser database to predict potential molecular targets of USP35. The results showed that STING was one of the most likely targets (Table S2). IHC staining confirmed that the expression of STING in GC tissues was positively correlated with USP35 (Fig. S4A, B), and high expression of STING indicated poor prognosis for GC patients (Fig. S4C, D). Subsequently, RT-qPCR showed that knocking down and overexpressing USP35 did not affect the mRNA level of STING (Fig. 3A). Western blot analysis indicated that the protein level of this protein in GC cells decreased after USP35 knockdown and increased after USP35 overexpression (Fig. 3B). To further investigate the underlying mechanism of this, we used the protein synthesis inhibitor CHX to evaluate the effect of USP35 on the stability of the STING protein. The results showed that USP35 overexpression increased the half-life of STING, while USP35 knockdown significantly decreased it (Fig. 3C). Treatment with the proteasome inhibitor MG132 reversed the decrease in STING in USP35-knockdown GC cells and promoted the accumulation of endogenous STING in USP35-overexpressing GC cells (Fig. 3D). These data suggest that USP35 interferes with the degradation of STING through the ubiquitin–proteasome system. Subsequently, co-immunoprecipitation showed an interaction between USP35 and STING in GC cells (Fig. 3E). Moreover, in USP35-knockdown GC cells, the ubiquitination level of STING was significantly increased, while the opposite was observed after USP35-overexpression, indicating that USP35 inhibits the ubiquitination of STING (Fig. 3F). Further, we found that USP35 WT but not USP35 C450A catalyzed STING de-ubiquitination (Fig. 3G). To explore the types of polyubiquitin connections to USP35-modified STING, we co-transfected STING with WT HA-ub and ubiquitin mutants. It was found that USP35 could remove K6-, K11-, K27-, K29-, K48, and k63-connected polyubiquitin chains from this protein (Fig. 3H). In summary, these data suggest that USP35 mediates de-ubiquitination by interacting with STING, thereby stabilizing its expression.

### USP35/STING promotes the adhesion of GC cells by activating the HIF-1α/FAK pathway

**Fig. 4:**
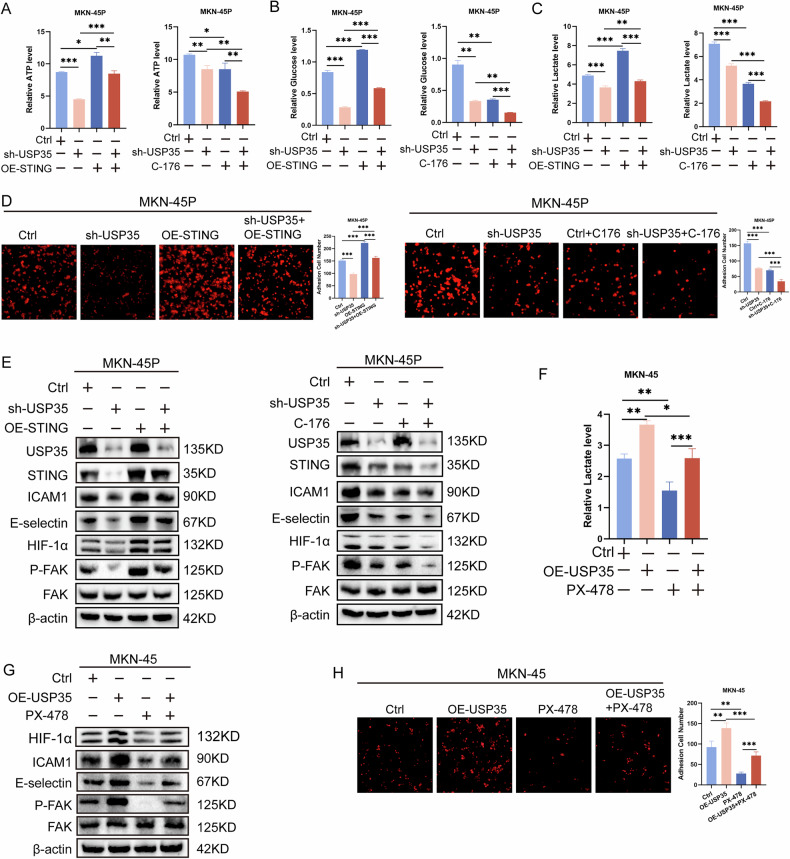
USP35/STING promotes peritoneal colonization of GC cells by activating the HIF-1α/FAK pathway. **A–C** The effects of sh-USP35, OE-STING, C-176 (STING inhibitor), and their combination on the metabolites in glycolysis (*n* = 3). **D** The effect of sh-USP35, OE-STING, C-176, and their combination on colonization ability of GC cells (*n* = 5); scale bar: 500μm. **E** Western blotting was used to detect expression adhesion molecules and the key protein of the HIF-1α/FAK pathway in GC cells by stimulating sh-USP35, OE-STING, C-176, and their combination. **F** Lactate production in GC cells in the presence of PX-478 (*n* = 3). **G** Protein expression adhesion molecules and the key protein of the HIF-1α/FAK pathway in GC cells in the presence of PX-478. **H**. The effect of the presence of PX-478 on the colonization ability of GC cells (*n* = 5); scale bar: 100μm. Data are presented as the mean ± SD. Gastric cancer, GC overexpression, OE short hairpin, sh. *P < 0.05; **P < 0.01; ***P < 0.001.

To determine whether USP35 expression promotes the colonization of GC cells by stabilizing STING expression, we simultaneously interfered with the expression of USP35 and STING (Fig. S2E, F). We found that compared with the USP35-knockdown group, the combination group of USP35 knockdown and STING overexpression could upregulate glucose uptake, lactate, and ATP levels in GC cells, while the combination group of USP35 knockdown and the STING inhibitor C-176 had the opposite effect (Fig. 4A–C). Adhesion assays showed that USP35 promoted the upregulation of colonization ability between GC cells and HMrSV5 by stabilizing STING expression (Fig. 4D), and Western blot analysis revealed that USP35 upregulated the expression of ICAM1 and e-selectin through the same stabilization (Fig. 4E).

Previous studies have shown that the HIF-1α/FAK signaling pathway can regulate cell adhesion [17]. Considering that STING can stabilize the expression of HIF-1α [11], we hypothesized that USP35/STING might regulate the adhesion of GC cells by activating the HIF-1α/FAK signaling pathway. The results showed that USP35 upregulated the expression of HIF-1α and p-FAK by stabilizing STING (Fig. 4E). Further treatment of USP35-overexpressing GC cells with PX-478 (HIF-1α inhibitor) showed that compared with the USP35-overexpression group, the combination group of USP35 overexpression and PX-478 had decreased lactate levels and decreased expression of ICAM1, e-selectin, HIF-1α, and p-FAK, as well as reduced colonization ability (Fig. 4F–H). These data confirm that USP35/STING promotes the colonization of GC cells by activating the HIF-1α/FAK pathway.

### Exosome USP35 enhances the adhesion ability of GC cells by promoting MMT in PMCs

**Fig. 5:**
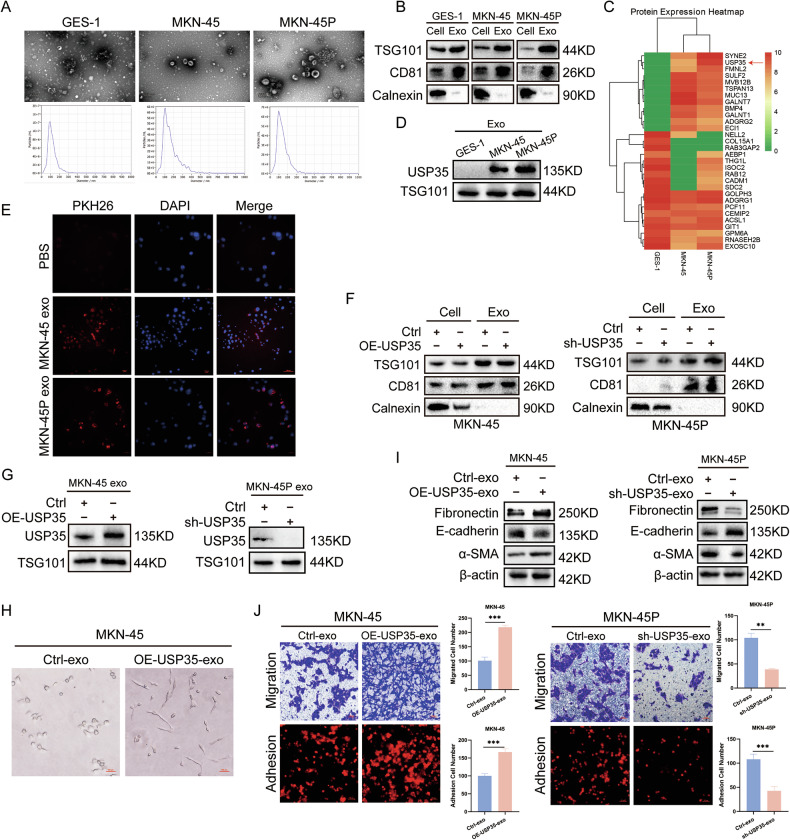
Exosome USP35 enhances the colonization ability of GC cells by promoting MMT in PMCs. **A** TEM and NTA analyzed the morphology and size of exosomes isolated from GES-1, MKN-45, and MKN-45P cell culture supernatants; scale bar: 200 nm. **B** Immunoblotting analyzed the purity of isolated exosomes by detecting the EV-specific markers CD81 and TSG101, with cytosolic protein calnexin as a negative control. **C** The label-free protein spectrum was used to detect the protein expression of exosomes. **D** Immunoblotting analyzed the background expression of USP35 in exosomes. **E** The internalization of PKH26-labeled exosomes secreted by GC cells was observed using an immunofluorescent microscope; scale bar: 200 μm. **F** Immunoblotting identified the purity of exosomes isolated from OE-USP35 and sh-USP35 gastric cancer cell culture supernatants. **G** Immunoblotting identified the overexpression and knockdown efficiency of USP35 in exosomes. **H** Morphological changes in HMrSV5 stimulated with OE-USP35 exosomes; scale bar: 500μm. **I** Immunoblotting found MMT-specific markers of HMrSV5 stimulated with OE-USP35 and sh-USP35 exosomes. **J** The effect of the presence of OE-USP35 and sh-USP35 exosomes on the colonization and migration ability of HMrSV5 (*n* = 5); scale bar: 500μm. Data are presented as the mean ± SD. Transmission electron microscope, TEM exosomes, Exo nanoparticle tracking analysis, NTA exosomal vesicle, EV overexpression, OE short hairpin, sh mesothelial–mesenchymal transition, MMT peritoneal mesothelial cell, PMC gastric cancer, GC. **P < 0.01; ***P < 0.001.

Exosomes promote MMT in the peritoneal mesothelial layer by upregulating the extracellular signal-regulated kinase pathway, creating a “soil” conducive to the attachment and growth of tumor cells. Therefore, we explored whether exosome proteins derived from GC cells could act on PMCs. First, we collected the culture supernatants of GES-1, MKN-45, and MKN-45P cells to extract exosomes and then performed electron microscopy observation and nanoparticle tracking analysis. As shown in Fig. 5A, typical cup-shaped structured particles were observed under both conditions. Western blot analysis confirmed that the expression of the exosome protein markers CD81 and TSG101 was significantly higher than that in the cell background (Fig. 5B). Further proteomic analysis revealed that the expression of USP35 protein in exosomes derived from GES-1, MKN-45, and MKN-45P showed a gradually increasing trend (Fig. 5C), which was also verified at the Western blot level (Fig. 5D); these results indicate that exosome USP35 is closely related to the high peritoneal metastasis characteristic of MKN-45P cells. We co-cultured HMrSV5 cells with DID-labeled exosomes derived from GC cells and PBS for 6 h to detect the internalization status of exosomes. The results showed that DID-labeled exosomes were effectively taken up by HMrSV5 cells (Fig. 5E). Subsequently, we extracted exosomes from USP35-overexpressing and USP35-knockdown GC cells to stimulate HMrSV5 cells (Fig. 5F, G). The results showed that exosome USP35 could change the morphology of HMrSV5 cells from round to long and spindle-shaped (Fig. 5H). Furthermore, it could upregulate the expression of fibronectin and α-SMA and downregulate the expression of E-cadherin in HMrSV5 cells (Fig. 5I). Transwell and adhesion assays showed that the migration and colonization abilities of HMrSV5 cells were enhanced after stimulation with exosome USP35 (Fig. 5J). These results indicate that exosome USP35 promotes MMT in PMCs, enabling tumor cells to better survive and proliferate upon attaching the peritoneum.

### USP35 promotes the adhesion and colonization of GC cells to PMCs, facilitating GCPD in vivo

**Fig. 6:**
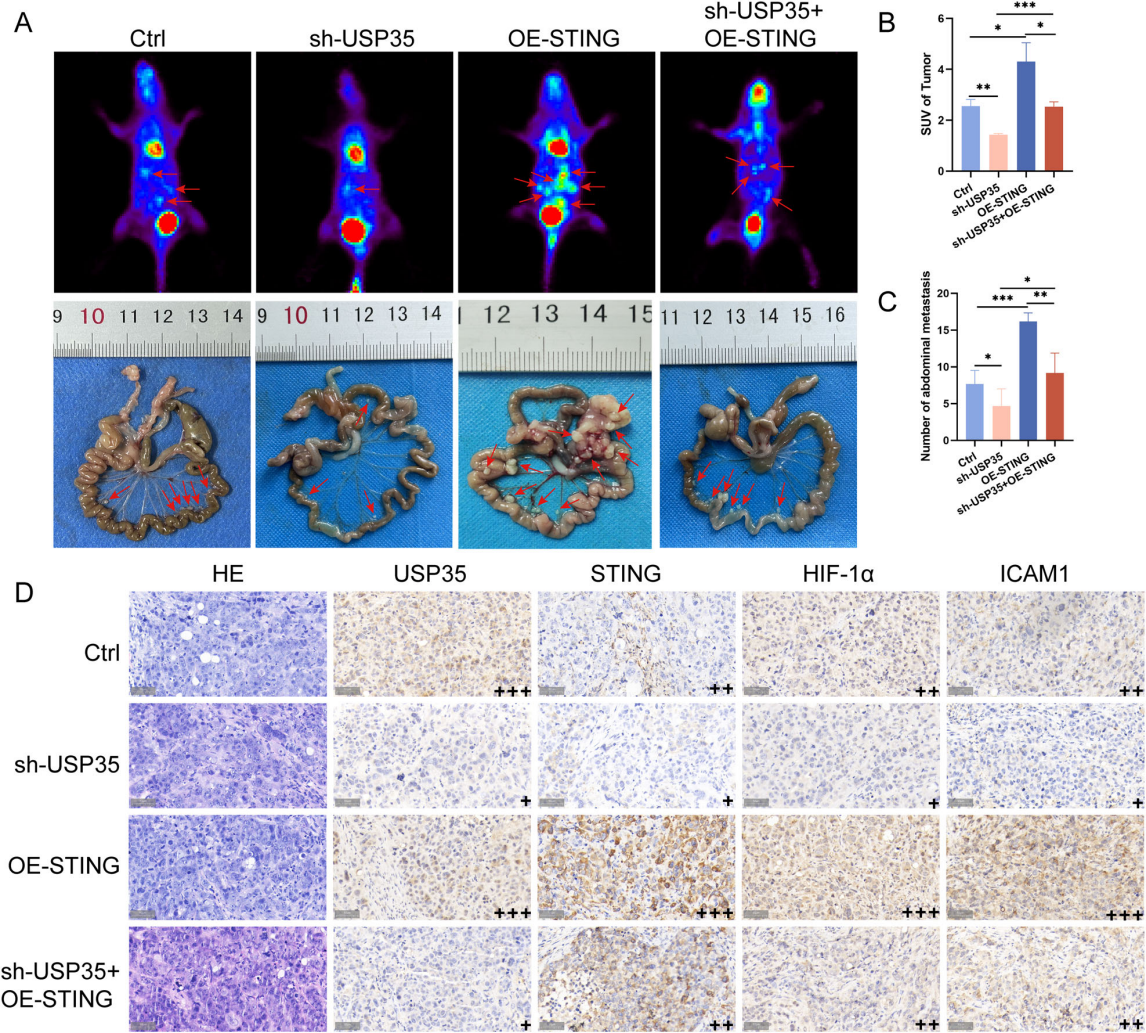
USP35 promotes GCPD by enhancing the adhesion ability of GC cells in vivo. **A** ^18^FDG micro-PET scans and photographs of abdominal disseminated tumors of the mice injected with sh-USP35 and/or OE-STING. **B** The SUVs of the abdominal disseminated tumors in vivo (mice per group, *n* = 6). **C** The number of abdominal disseminated tumors (mice per group, *n* = 6). **D** Xenograft tumor tissue was evaluated via HE and IHC (×400); scale bar: 50 μm. Data are presented as the mean ± SD. Hematoxylin and eosin staining, HE; immunohistochemistry, IHC; gastric cancer, GC; standard uptake value, SUV. *P < 0.05; **P < 0.01; ***P < 0.001.

To validate the conclusions drawn from GC-cell-level studies, we established a peritoneal dissemination model. ^18^FDG micro-PET scanning was used to detect the degree of peritoneal dissemination after interfering with USP35 expression (Fig. 6A). Compared with the control group, treatment with USP35 knockdown reduced the tumor standard uptake value (SUV). The combined treatment group of USP35 knockdown and STING overexpression alleviated the increase in glucose uptake caused by the overexpression (Fig. 6A, B). Similarly, after sacrificing the mice, the combined use of USP35 knockdown and STING overexpression reversed the tumor dissemination promoted by the overexpression (Fig. 6C). IHC staining was performed to detect the expression of USP35, STING, HIF-1α, and ICAM1 in the tumor tissues, and the results were consistent with those obtained in vitro (Fig. 6D). Specifically, in GC cells, the de-ubiquitinase USP35 activates the HIF-1α/FAK signaling pathway by stabilizing the expression of STING, which in turn regulates energy metabolic reprogramming, thereby promoting GCPD.

**Fig. 7:**
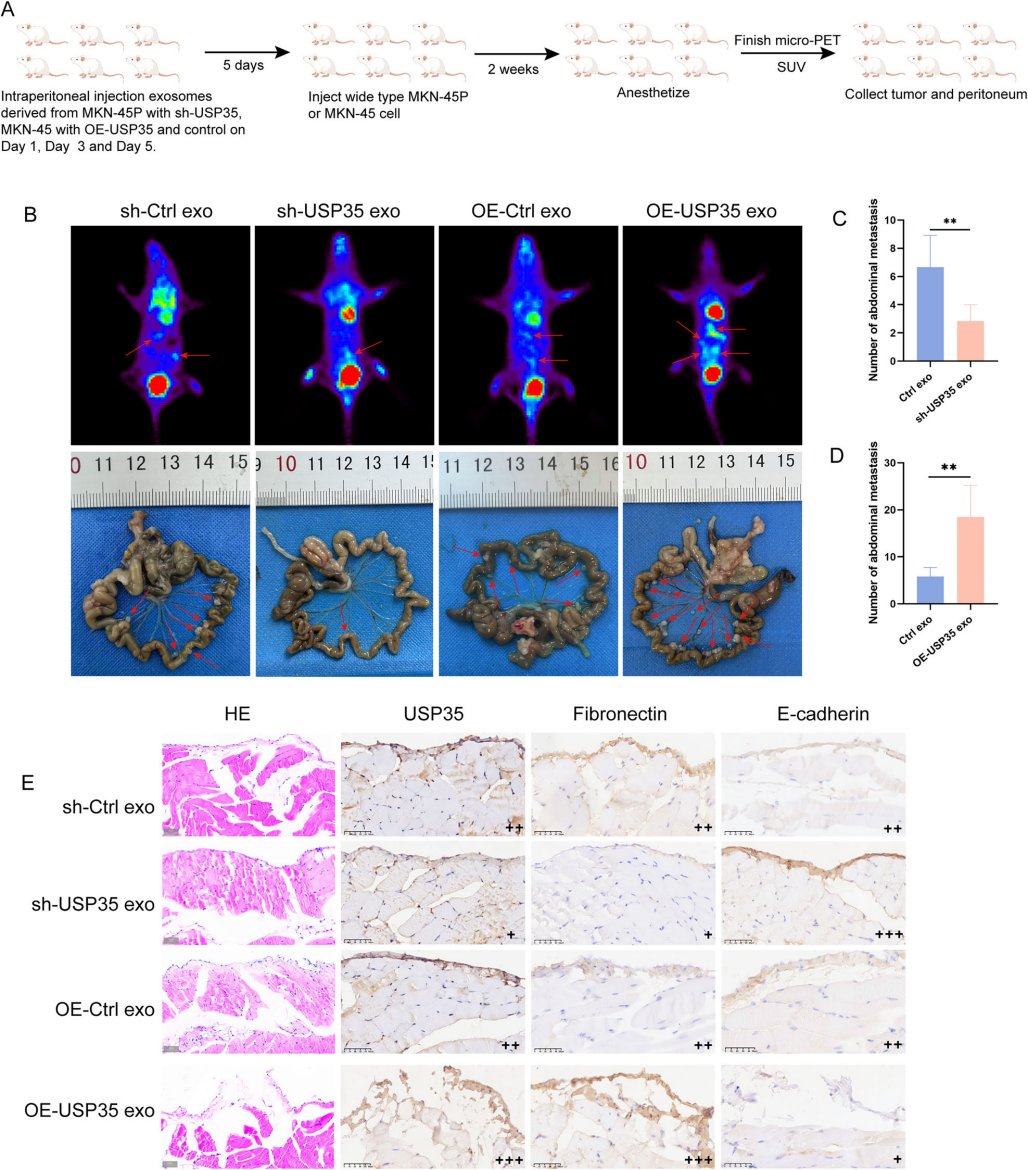
Exosome USP35 derived from GC cells promotes GCPD by inducing MMT of PMCs in vivo. **A** A flow diagram of the construction of the mouse model to identify the role of exosome USP35. **B** ^18^FDG micro-PET scans and photographs of abdominal disseminated tumors of the mice stimulated with OE-USP35 and sh-USP35 exosomes and injected with wild-type GC cells. **C, D** The number of abdominal disseminated tumors (mice per group, *n* = 6). **E** Peritoneal tissue was evaluated via HE and IHC; scale bar: 50μm. Data are presented as the mean ± SD. Hematoxylin and eosin staining, HE immunohistochemistry, IHC gastric cancer, GC. *P < 0.05; **P < 0.01; ***P < 0.001.

To validate the effect of exosome USP35 on PMCs, we first stimulated the abdominal cavity with exosomes for 5 days and then established a peritoneal dissemination model using wild-type GC cells (Fig. 7A). ^18^FDG micro-PET scanning was used to detect the degree of peritoneal dissemination after stimulation with exosome USP35. Compared with the control group, treatment with exosome USP35 promoted tumor dissemination (Fig. 7B–D). HE staining revealed that it also disrupted the continuity of the peritoneal mesothelium in mice. IHC staining was used to detect the expression of USP35, E-cadherin, and fibronectin in the peritoneum, and the results were consistent with those obtained in vitro (Fig. 7E). These data indicate that exosome USP35 derived from GC cells promotes MMT in PMCs, providing a favorable environment for the adhesion and colonization of GC cells on the peritoneum and thus facilitating GCPD.

## Discussion

In 1889, Stephen Paget proposed the “seed and soil” theory, pointing out that metastasis depends on the interaction between the seed (tumor cells) and the soil (the microenvironment), which highlights the importance of the tumor microenvironment [18]. Some studies have found that tumor cells can induce the formation of microenvironments in distant organs, which is beneficial to their survival and growth after they reach specific organs [19, 20]. These microenvironments prepared in advance are called “pre-metastatic niches (PMNs)”. Not all disseminated GC cells can successfully form peritoneal metastases. This indicates that GC cells with certain characteristics and the pre-metastatic microenvironment in the peritoneum together determine the ability of these cells to select peritoneal organs for directional metastasis. This study confirms that USP35 de-ubiquitinates STING and activates the HIF-1α/FAK signaling pathway, resulting in the reprogramming of energy metabolism in GC cells and thereby enhancing their adhesion ability. At the same time, exosome USP35 derived from GC cells induces MMT in PMCs, induces the formation of peritoneal-specific PMNs, and creates favorable conditions for the attachment and growth of tumor cells in the peritoneum.

Some studies have confirmed that patients with high USP35 expression in ovarian cancer, liver cancer, clear cell renal cell carcinoma, and cutaneous melanoma have a poor prognosis [16, 21–23]. Similarly, in this study, both bioinformatics analysis and IHC confirmed that with the progression of gastric diseases, the expression of USP35 increases successively in adjacent normal tissues, GC tissues, and GC tissues with peritoneal metastasis. The analysis of clinicopathological parameters suggests that high USP35 expression is positively correlated with peritoneal metastasis of gastric cancer and also predicts a poor prognosis for patients. At the cellular level, USP35 was most highly expressed in MKN-45P cells, suggesting that it plays an important role in the high peritoneal metastasis ability of MKN-45P. This indicates that USP35 has the potential to become a diagnostic and prognostic biomarker for peritoneal metastasis of gastric cancer.

Changes in the state of cell–cell and cell–matrix adhesion make cancer cells more viable during the multi-step cascade of cancer progression, enabling them to degrade the extracellular matrix, survive in the circulation, and colonize at distant metastatic sites. Importantly, metabolic reprogramming of aerobic glycolysis promotes the colonization of cancer cells in the peritoneum by providing energy for their adhesion [24, 25]. In this study, GSEA demonstrated that USP35 was positively correlated with energy metabolism and cell adhesion in GC. In addition, we observed that USP35 enhanced the adhesion between GC cells and PMCs by promoting glycolysis. Specifically, we found that overexpression of USP35 could promote cellular lactate production, glucose uptake, ATP levels, and cell adhesion. After adding 2-DG to inhibit glycolysis, the enhanced cell adhesion ability caused by the overexpression of USP35 could be reversed. It has been reported that glycolysis increases the accumulation of reactive oxygen species and the activation of p38 MAPK, leading to the activation and adhesion of monocytes [26], which also supports the idea that glycolysis can promote cell adhesion. Some studies have also reported that USP35 promotes the progression of hepatocellular carcinoma by protecting the key glycolytic enzyme PKM2 from ubiquitination-mediated degradation [21]. Additionally, this protein promotes the progression of breast cancer by regulating the ubiquitination of PFK-1 and mediating glycolysis [27]. The above-mentioned studies confirm that USP35 can promote glycolysis, which is consistent with our conclusion. In conclusion, identifying the oncogenic signals responsible for the reprogramming of glucose metabolism may be translated into better treatments against peritoneal metastasis.

Furthermore, this study found that USP35 can directly de-ubiquitinate STING; the combined group of USP35 knockdown and STING overexpression could alleviate the glycolysis and adhesion inhibition caused by the knockdown. Literature reports have also confirmed that in ovarian cancer, USP35 can directly de-ubiquitinate and inactivate STING, thereby enhancing the chemoresistance of ovarian cancer cells to chemotherapy drugs and promoting tumor development [16]. Subsequently, we confirmed that USP35/STING can increase the expression of HIF-1α and P-FAK. Moreover, the HIF-1α inhibitor (PX-478) can reverse the increase in P-FAK and the enhanced adhesion ability caused by the overexpression of USP35. As is well known, high expression of HIF-1α enhances cellular glycolysis and promotes tumor progression [28]. Focal adhesion kinase (FAK) regulates the dynamic changes in focal adhesions through its own phosphorylation and binding to other proteins, enabling it to stably anchor cells, maintain their adhesion state, and promote tumor progression [29]. Previous studies have also shown that HIF-1α can inhibit the migration and adhesion of smooth muscle cells by regulating the activity of FAK [17]. The above-mentioned findings confirm that HIF-1α can induce glycolysis and activate FAK, thereby enhancing cell adhesion, which also supports our conclusion that USP35/STING promotes glycolysis and further enhances cell adhesion by activating the HIF-1α/FAK signaling pathway.

GC-cell-derived exosomes may be involved in the pathological process of peritoneal dissemination by mediating the crosstalk between cancer cells and stromal cells, thus inducing enhanced tumor growth, migration, adhesion, and invasion abilities; peritoneal fibrosis and apoptosis; MMT; angiogenesis; and chemoresistance [30]. In the process of the formation of peritoneal metastasis, the adhesion between GC cells and the inner layer of the peritoneum is extremely crucial. Metastasis through the mesothelium is an important mode of this [31–33]. In the process of mesothelium-mediated metastasis, cancer cells disseminated in the peritoneal cavity directly adhere to the surface of the peritoneum [34]. Therefore, creating a favorable pre-metastatic microenvironment in the peritoneal mesothelial layer and disrupting the defense of the basement membrane are two key factors for achieving peritoneal metastasis. For the first time, our study used exosome proteomics to find that exosome USP35 is significantly highly expressed in MKN-45P cells and significantly correlated with the high peritoneal metastasis of MKN-45P. It also promotes the MMT of PMCs, which is beneficial to the adhesion and colonization of GC cells. In vivo verification also showed that exosome USP35 disrupted the continuity of the peritoneal mesothelium, facilitating peritoneal dissemination in mice. Given the crucial role of exosome USP35 in promoting the peritoneal metastasis of gastric cancer cells, it is expected to become a potential diagnostic biomarker. By detecting its levels in GC patients, we may be able to predict the risk of peritoneal metastasis in advance so as to take more active and effective intervention measures.

In conclusion, for the first time, we discovered that in GC cells, USP35 can de-ubiquitinate STING, activate the HIF-1α/FAK pathway, and promote the adhesion and colonization of GC cells to PMCs. Meanwhile, exosome USP35 derived from GC cells induces MMT in PMCs, forms peritoneal-specific PMNs, and promotes adhesion. This study provides a new theoretical basis for a deeper understanding of the mechanism of GCPD and helps to provide new targets and ideas for the research and development of strategies for the prevention and treatment of GC, especially GCPD.

## Supplementary information

Supplementary materials

Western blot

## Data Availability

The original contributions presented in this study are included in the article/supplementary material, further inquiries can be directed to the corresponding author.
