# Supplementary material for: De-ubiquitinase USP35 promotes peritoneal dissemination of gastric cancer by regulating metabolic reprogramming
Source: Cell Death Dis. 2025 Dec 10;16(1):889. doi: 10.1038/s41419-025-08322-4 (PMC12706015; doi:10.1038/s41419-025-08322-4)
Supplement: Supplementary file 1 — Supplementary materials [file 41419_2025_8322_MOESM1_ESM.docx]

| **Table S1. The primer sequence used for qRT-PCR** | |
| --- | --- |
| **Primer name** | **sequence** |
| **USP35** |  |
| F | TCGAATCTGTCAGCAACGTC |
| R | TGTCTTTGGAAATGGCTTCC |
| **STING** |  |
| F | CCAGAGCACACTCTCCGGTA |
| R | CGCATTTGGGAGGGAGTAGTA |
| **β-actin** |  |
| F | ATGTGGCCGAGGACTTTGATT |
| R | AGTGGGGTGGCTTTTAGGATG |
|  |  |

| **Table S2. The potential deubiquitization targets of USP35 by UbiBrowser database.** | | | | | | | | | |
| --- | --- | --- | --- | --- | --- | --- | --- | --- | --- |
| **SwissProt ID (DUB)** | **Gene Symbol (DUB)** | **SwissProt ID (Substrate)** | **Gene Symbol (Substrate)** | **Domain_LikelihoodRatio** | **Go_LikelihoodRatio** | **Network_LikelihoodRatio** | **Motif_LikelihoodRatio** | **Confidence Score** | **Species** |
| Q9P2H5 | USP35 | P04637 | TP53 | 7.88 | 3.63 | 1 | 9.23 | 0.918 | H.sapiens |
| **Q9P2H5** | **USP35** | **Q86WV6** | **STING1** | **7.88** | **2.2** | **1** | **5.68** | **0.88** | **H.sapiens** |
| Q9P2H5 | USP35 | P45880 | VDAC2 | 4.24 | 2.2 | 1 | 4.28 | 0.832 | H.sapiens |
| Q9P2H5 | USP35 | Q96RL1 | UIMC1 | 4.24 | 1.84 | 1 | 4.28 | 0.821 | H.sapiens |
| Q9P2H5 | USP35 | Q9Y4K3 | TRAF6 | 4.24 | 1.99 | 1 | 5.68 | 0.843 | H.sapiens |
| Q9P2H5 | USP35 | Q6XPS3 | TPTE2 | 4.24 | 2.2 | 1 | 4.09 | 0.829 | H.sapiens |
| Q9P2H5 | USP35 | O15350 | TP73 | 4.24 | 1.84 | 1 | 4.28 | 0.821 | H.sapiens |
| Q9P2H5 | USP35 | Q9H3D4 | TP63 | 4.24 | 1.84 | 1 | 4.28 | 0.821 | H.sapiens |
| Q9P2H5 | USP35 | Q92922 | SMARCC1 | 4.24 | 1.48 | 1 | 4.28 | 0.807 | H.sapiens |
| Q9P2H5 | USP35 | P60484 | PTEN | 4.24 | 2.2 | 1 | 5.68 | 0.849 | H.sapiens |
| Q9P2H5 | USP35 | Q14863 | POU6F1 | 4.24 | 1 | 1 | 9.23 | 0.831 | H.sapiens |
| Q9P2H5 | USP35 | Q01860 | POU5F1 | 4.24 | 1.48 | 1 | 4.28 | 0.807 | H.sapiens |
| Q9P2H5 | USP35 | Q12837 | POU4F2 | 4.24 | 1.48 | 1 | 9.23 | 0.854 | H.sapiens |
| Q9P2H5 | USP35 | Q01851 | POU4F1 | 4.24 | 1.48 | 1 | 9.23 | 0.854 | H.sapiens |
| Q9P2H5 | USP35 | P20264 | POU3F3 | 4.24 | 1 | 1 | 9.23 | 0.831 | H.sapiens |
| Q9P2H5 | USP35 | P20265 | POU3F2 | 4.24 | 1 | 1 | 9.23 | 0.831 | H.sapiens |
| Q9P2H5 | USP35 | Q9Y446 | PKP3 | 4.24 | 1.84 | 1 | 4.09 | 0.818 | H.sapiens |
| Q9P2H5 | USP35 | P04198 | MYCN | 4.24 | 1.48 | 1 | 4.28 | 0.807 | H.sapiens |
| Q9P2H5 | USP35 | P12524 | MYCL | 4.24 | 1.48 | 1 | 9.23 | 0.854 | H.sapiens |
| Q9P2H5 | USP35 | P01106 | MYC | 4.24 | 1.48 | 1 | 5.68 | 0.825 | H.sapiens |
| Q9P2H5 | USP35 | O15151 | MDM4 | 4.24 | 1.48 | 1 | 4.28 | 0.807 | H.sapiens |
| Q9P2H5 | USP35 | Q00987 | MDM2 | 4.24 | 2.2 | 1 | 5.68 | 0.849 | H.sapiens |
| Q9P2H5 | USP35 | P27338 | MAOB | 4.24 | 1.48 | 1 | 4.28 | 0.807 | H.sapiens |
| Q9P2H5 | USP35 | Q8NB78 | KDM1B | 4.24 | 1.84 | 1 | 4.28 | 0.821 | H.sapiens |
| Q9P2H5 | USP35 | O60341 | KDM1A | 4.24 | 1.84 | 1 | 5.68 | 0.838 | H.sapiens |
| Q9P2H5 | USP35 | P14923 | JUP | 4.24 | 1.84 | 1 | 5.68 | 0.838 | H.sapiens |
| Q9P2H5 | USP35 | Q9Y6K9 | IKBKG | 4.24 | 2.2 | 1 | 4.09 | 0.829 | H.sapiens |
| Q9P2H5 | USP35 | P35222 | CTNNB1 | 4.24 | 1.84 | 1 | 5.68 | 0.838 | H.sapiens |
| Q9P2H5 | USP35 | P10275 | AR | 4.24 | 2.2 | 1 | 9.23 | 0.874 | H.sapiens |
| Q9P2H5 | USP35 | O95996 | APC2 | 4.24 | 1.48 | 1 | 9.23 | 0.854 | H.sapiens |
| Q9P2H5 | USP35 | Q15911 | ZFHX3 | 2.88 | 1.48 | 1 | 9.23 | 0.831 | H.sapiens |
| Q9P2H5 | USP35 | Q01831 | XPC | 2.88 | 2.2 | 1 | 5.68 | 0.826 | H.sapiens |
| Q9P2H5 | USP35 | Q6EMK4 | VASN | 2.88 | 1.48 | 1 | 9.23 | 0.831 | H.sapiens |
| Q9P2H5 | USP35 | Q15672 | TWIST1 | 2.88 | 1.48 | 1 | 9.23 | 0.831 | H.sapiens |
| Q9P2H5 | USP35 | Q9HCS4 | TCF7L1 | 2.88 | 1.48 | 1 | 9.23 | 0.831 | H.sapiens |
| Q9P2H5 | USP35 | Q15542 | TAF5 | 2.88 | 1.48 | 1 | 9.23 | 0.831 | H.sapiens |
| Q9P2H5 | USP35 | O95343 | SIX3 | 2.88 | 1.99 | 1 | 9.23 | 0.849 | H.sapiens |
| Q9P2H5 | USP35 | Q9BYB0 | SHANK3 | 2.88 | 1.48 | 1 | 9.23 | 0.831 | H.sapiens |
| Q9P2H5 | USP35 | Q9Y566 | SHANK1 | 2.88 | 1.48 | 1 | 9.23 | 0.831 | H.sapiens |
| Q9P2H5 | USP35 | P37088 | SCNN1A | 2.88 | 1.48 | 1 | 9.23 | 0.831 | H.sapiens |
| Q9P2H5 | USP35 | Q13546 | RIPK1 | 2.88 | 2.2 | 1 | 4.28 | 0.807 | H.sapiens |
| Q9P2H5 | USP35 | Q96S59 | RANBP9 | 2.88 | 1.48 | 1 | 9.23 | 0.831 | H.sapiens |
| Q9P2H5 | USP35 | P53350 | PLK1 | 2.88 | 1.84 | 1 | 9.23 | 0.844 | H.sapiens |
| Q9P2H5 | USP35 | Q8IXF0 | NPAS3 | 2.88 | 1.48 | 1 | 9.23 | 0.831 | H.sapiens |
| Q9P2H5 | USP35 | Q99583 | MNT | 2.88 | 1.48 | 1 | 9.23 | 0.831 | H.sapiens |
| Q9P2H5 | USP35 | P31260 | HOXA10 | 2.88 | 1.48 | 1 | 9.23 | 0.831 | H.sapiens |
| Q9P2H5 | USP35 | Q5QNW6 | HIST2H2BF | 2.88 | 1.48 | 1 | 9.23 | 0.831 | H.sapiens |
| Q9P2H5 | USP35 | Q16778 | HIST2H2BE | 2.88 | 1.48 | 1 | 9.23 | 0.831 | H.sapiens |
| Q9P2H5 | USP35 | Q6DRA6 | HIST2H2BD | 2.88 | 1.48 | 1 | 9.23 | 0.831 | H.sapiens |
| Q9P2H5 | USP35 | Q6DN03 | HIST2H2BC | 2.88 | 1.48 | 1 | 9.23 | 0.831 | H.sapiens |
| Q9P2H5 | USP35 | P23527 | HIST1H2BO | 2.88 | 1.48 | 1 | 9.23 | 0.831 | H.sapiens |
| Q9P2H5 | USP35 | Q93079 | HIST1H2BH | 2.88 | 1.48 | 1 | 9.23 | 0.831 | H.sapiens |
| Q9P2H5 | USP35 | Q03014 | HHEX | 2.88 | 1.48 | 1 | 9.23 | 0.831 | H.sapiens |
| Q9P2H5 | USP35 | Q5TGS1 | HES3 | 2.88 | 1.48 | 1 | 9.23 | 0.831 | H.sapiens |
| Q9P2H5 | USP35 | Q14469 | HES1 | 2.88 | 1.48 | 1 | 9.23 | 0.831 | H.sapiens |
| Q9P2H5 | USP35 | P57053 | H2BFS | 2.88 | 1.48 | 1 | 9.23 | 0.831 | H.sapiens |
| Q9P2H5 | USP35 | O60814 | H2BC12 | 2.88 | 1.48 | 1 | 9.23 | 0.831 | H.sapiens |
| Q9P2H5 | USP35 | P06899 | H2BC11 | 2.88 | 1.48 | 1 | 9.23 | 0.831 | H.sapiens |
| Q9P2H5 | USP35 | P62807 | H2BC10 | 2.88 | 1.48 | 1 | 9.23 | 0.831 | H.sapiens |
| Q9P2H5 | USP35 | O15399 | GRIN2D | 2.88 | 1.48 | 1 | 9.23 | 0.831 | H.sapiens |
| Q9P2H5 | USP35 | Q9H461 | FZD8 | 2.88 | 1.48 | 1 | 9.23 | 0.831 | H.sapiens |
| Q9P2H5 | USP35 | P35637 | FUS | 2.88 | 1.84 | 1 | 9.23 | 0.844 | H.sapiens |
| Q9P2H5 | USP35 | Q12948 | FOXC1 | 2.88 | 1.48 | 1 | 9.23 | 0.831 | H.sapiens |
| Q9P2H5 | USP35 | Q13202 | DUSP8 | 2.88 | 1.48 | 1 | 9.23 | 0.831 | H.sapiens |
| Q9P2H5 | USP35 | Q9H2U1 | DHX36 | 2.88 | 1.84 | 1 | 9.23 | 0.844 | H.sapiens |
| Q9P2H5 | USP35 | Q8TD26 | CHD6 | 2.88 | 1.48 | 1 | 9.23 | 0.831 | H.sapiens |
| Q9P2H5 | USP35 | A9YTQ3 | AHRR | 2.88 | 1.48 | 1 | 9.23 | 0.831 | H.sapiens |
| Q9P2H5 | USP35 | P35869 | AHR | 2.88 | 2.2 | 1 | 4.28 | 0.807 | H.sapiens |
| Q9P2H5 | USP35 | P55196 | AFDN | 2.88 | 1.48 | 1 | 9.23 | 0.831 | H.sapiens |


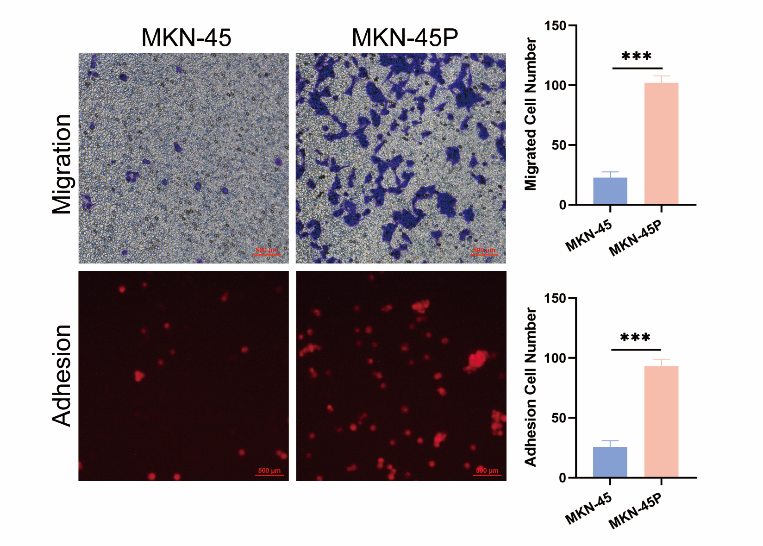


Figure S1. Transwell and adhesion assay identify the peritoneal metastasis potential between MKN-45 and MKN-45P (n = 5), scale bar: 500μm.


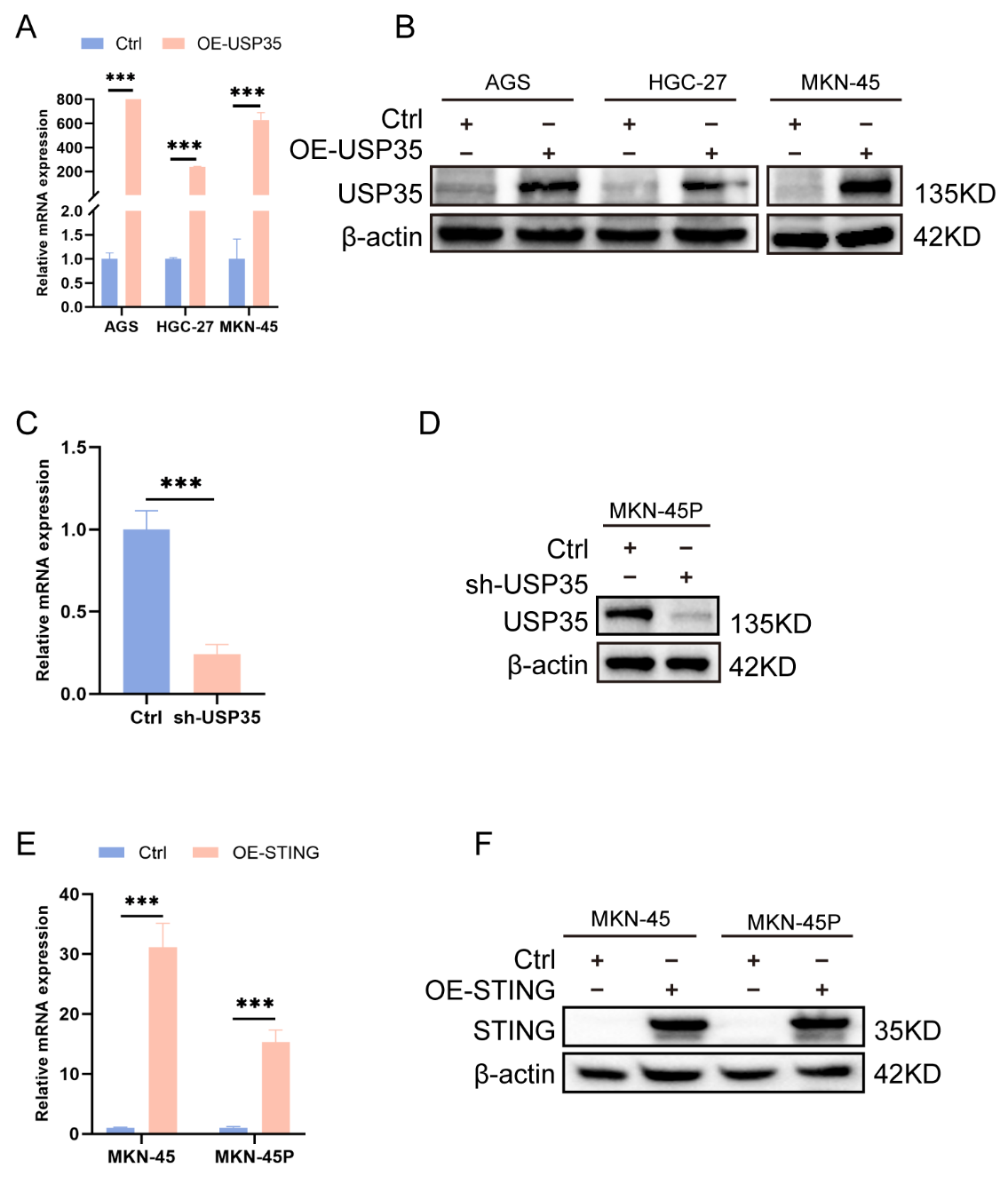


**Figure S2. Determination of transfection efficiency.** (A-B). qRT-PCR and Western blot were used to identify the overexpression rate of USP35 in AGS, HGC-27 and MKN-45 cell lines, respectively. (C-D). qRT-PCR and Western blot were used to identify the knockdown rate of USP35 in MKN-45P, respectively. (E-F). qRT-PCR and Western blot were used to identify the overexpression rate of OE-STING, respectively. qRT-PCR, quantitative real-time PCR. **P*<0.05, ***P*<0.01, ****P*<0.001.


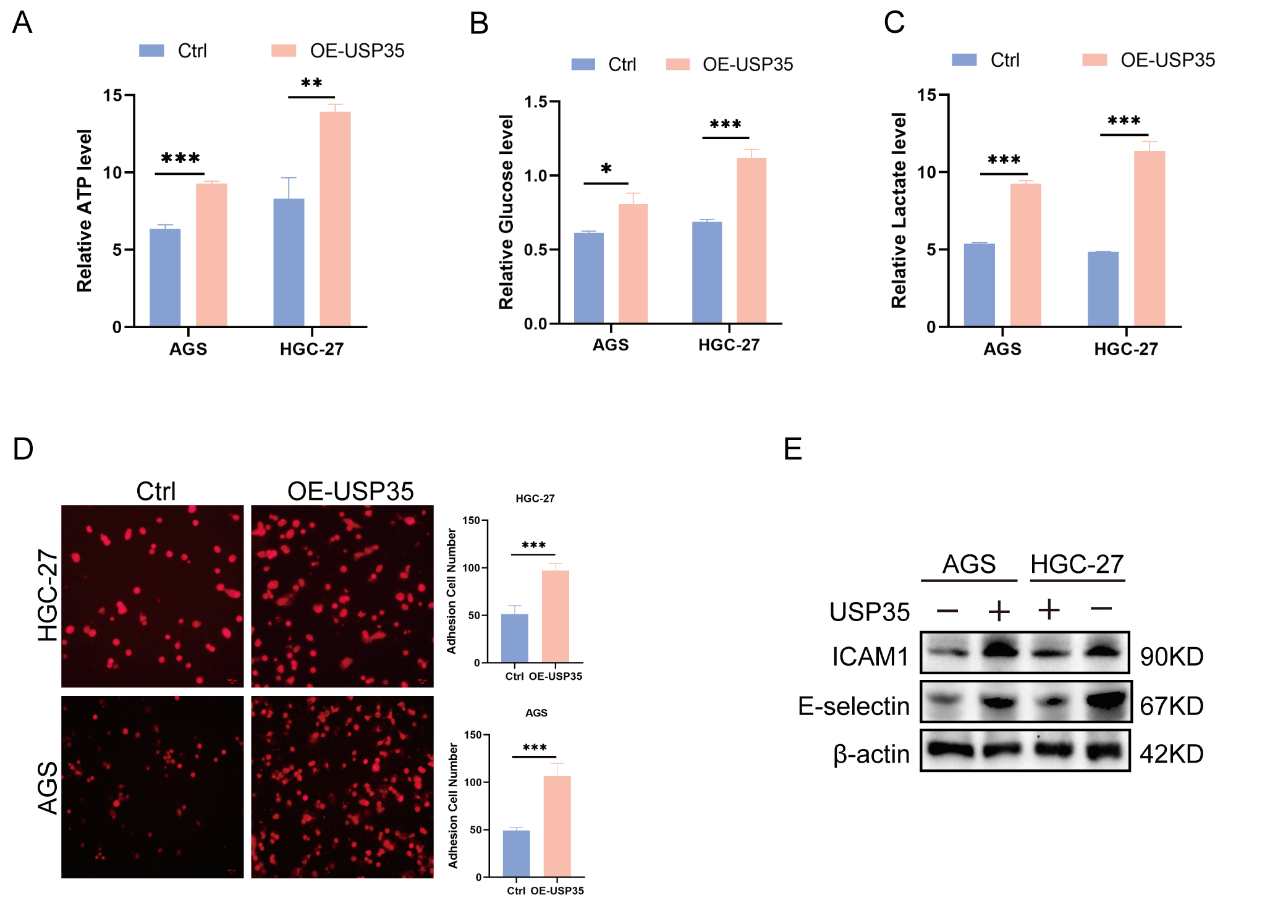


**Figure S3.** **USP35 regulates energy metabolism reprogramming and enhances the colonization ability of GC cells.** (A-C). The respective effects of USP35 on the metabolites in glycolysis in AGS and HGC-27. (D-E). Adhesion assay and Western blot disclosed that effect of USP35 on colonization ability of GC cells, scale bar: 100μm. **P*<0.05, ***P*<0.01, ****P*<0.001.


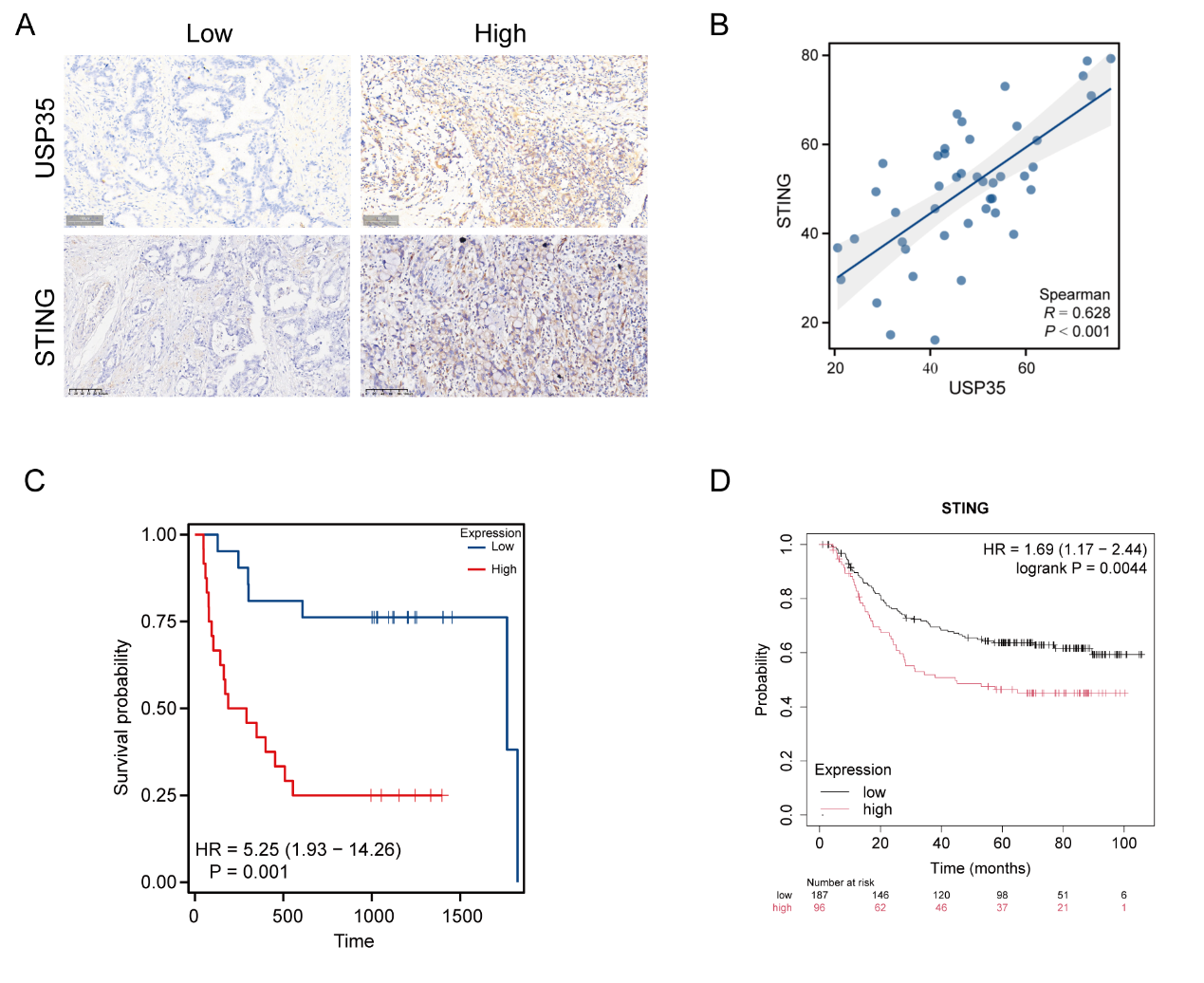


**Figure S4. The expression characteristics of STING in gastric cancer tissues.** (A). Immunohistochemical staining was used to detect the expression of STING in GC tissues (n=40), scale bar: 100μm. (B). The correlation analysis of USP35 and STING in GC tissues. (C). Kaplan-Meier survival analysis of the association of USP35 expression with OS in GC tissues. (D). GSE62254 was used to evaluate the association of STING expression with GC prognosis via Kaplan-Meier analysis. Gastric cancer, GC; Overall survival, OS.
