# Supplementary material for: De-ubiquitinase USP35 promotes peritoneal dissemination of gastric cancer by regulating metabolic reprogramming
Source: Cell Death Dis. 2025 Dec 10;16(1):889. doi: 10.1038/s41419-025-08322-4 (PMC12706015; doi:10.1038/s41419-025-08322-4)

Figure 2B：

USP35

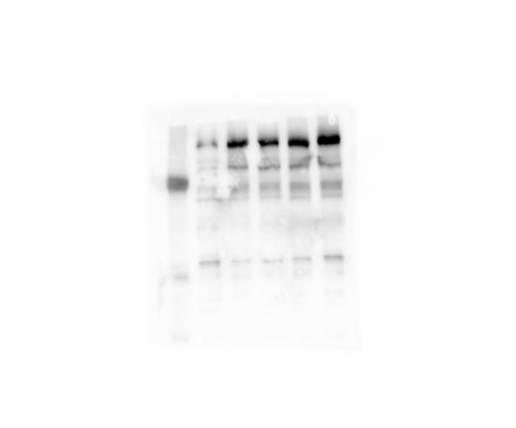

Actin

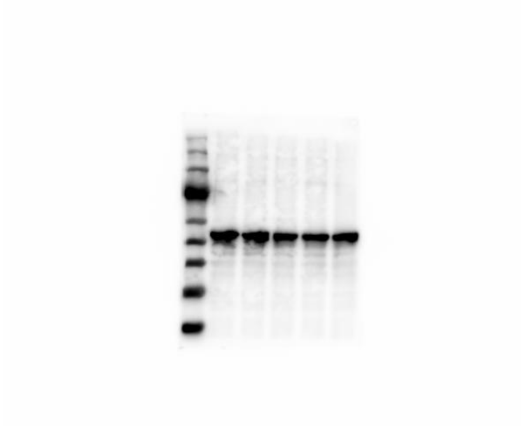

Figure 2I：MKN-45

e-selectin

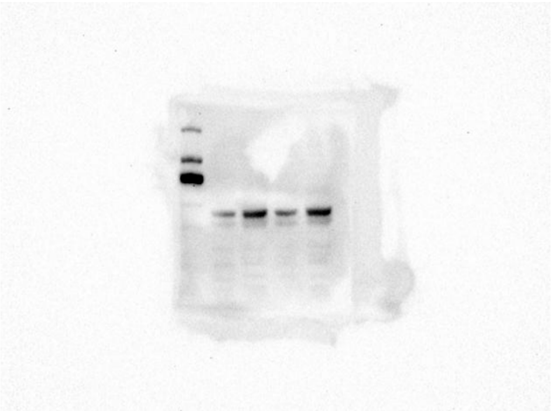

ICAM1

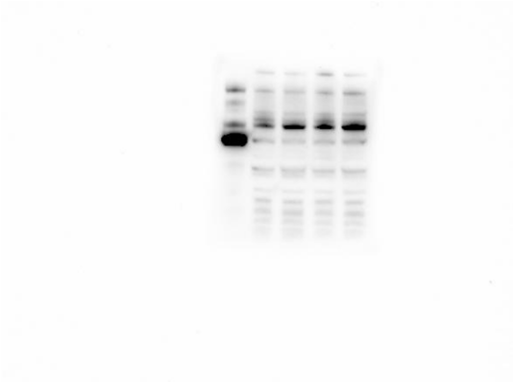

Actin

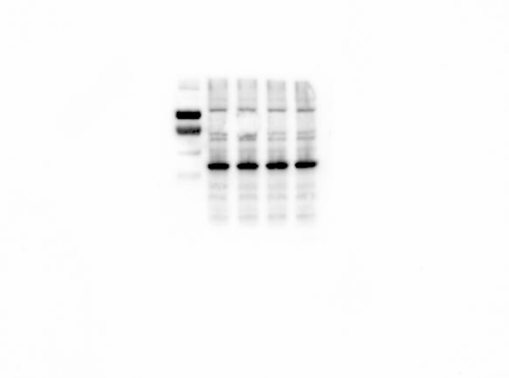

MKN-45P:

e-selectin

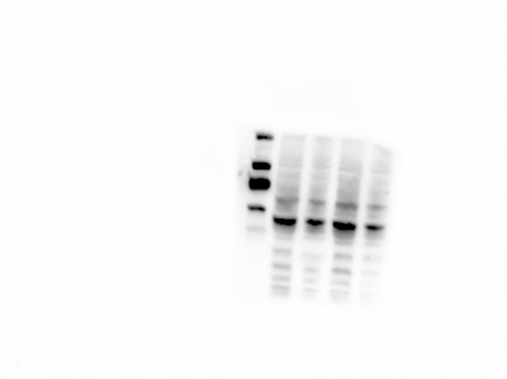

ICAM1

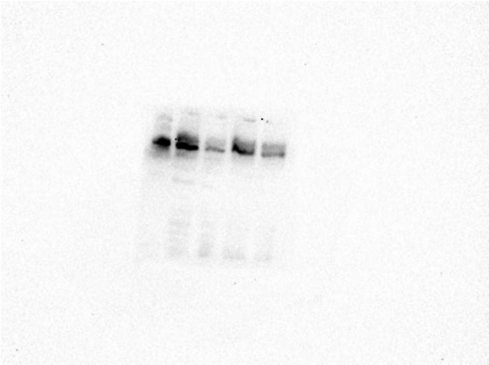

actin

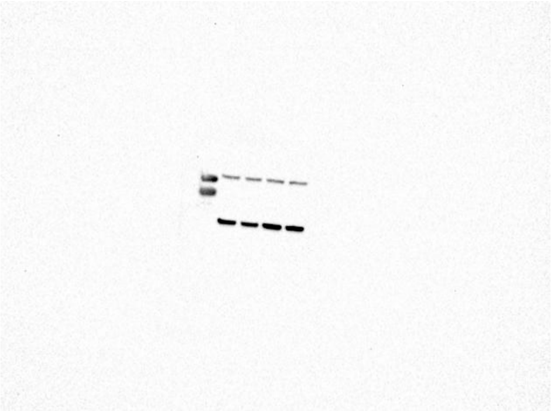

Figure 2L: MKN-45

e-selectin

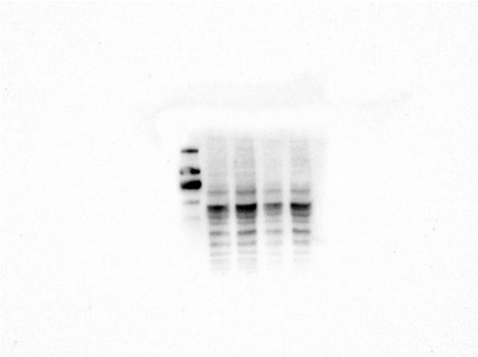

ICAM1

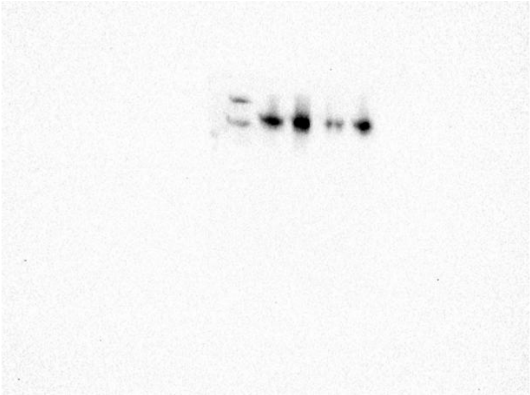

Actin

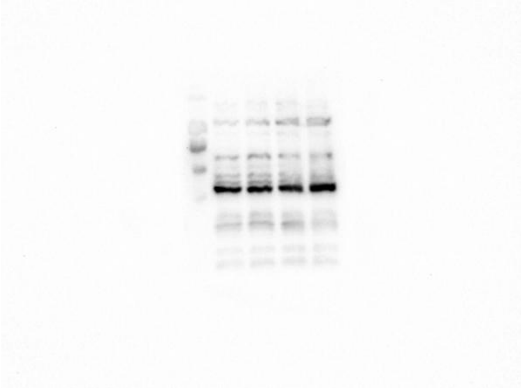

MKN-45P：

e-selectin

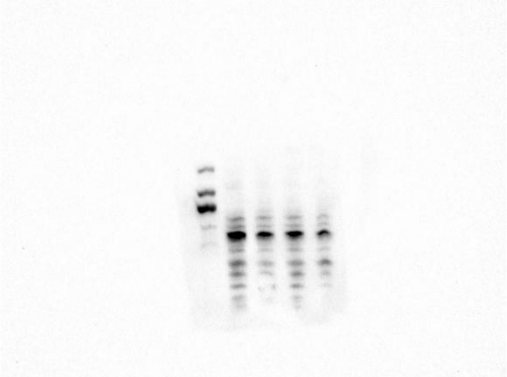

ICAM1

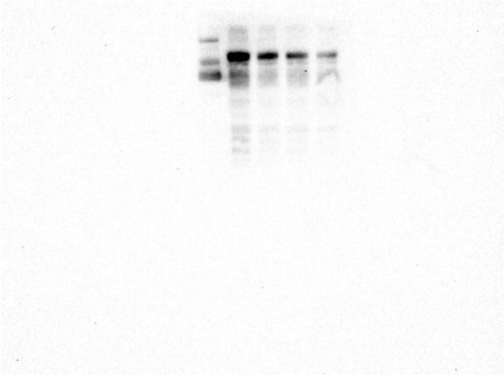

Actin

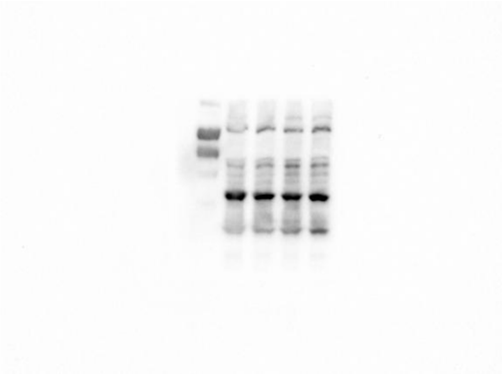

Figure 3B： MKN-45

STING

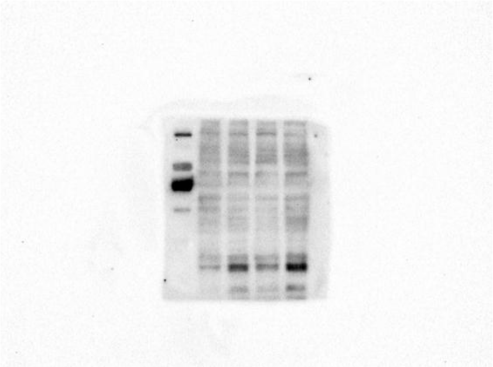

Actin

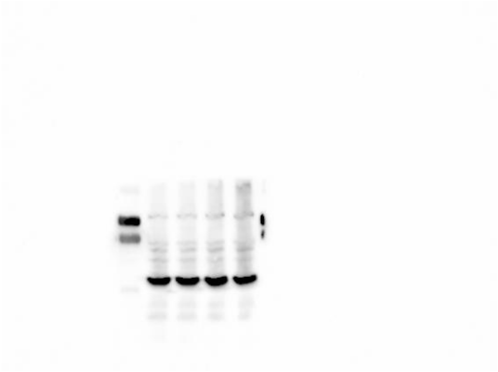

MKN-45P

STING

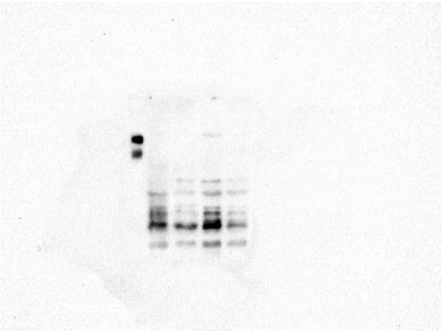

Actin

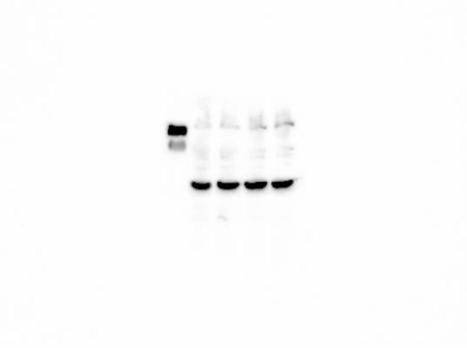

Figure 3C: MKN-45

USP35

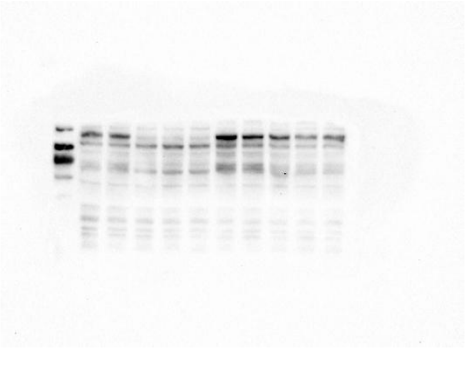

STING

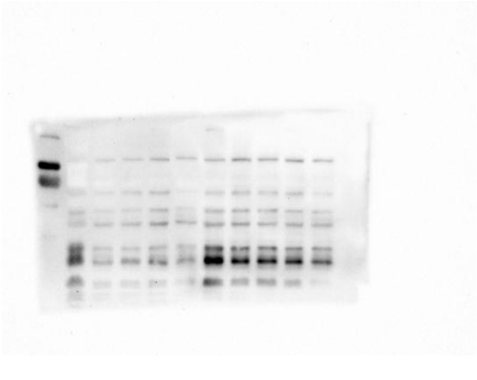

Actin

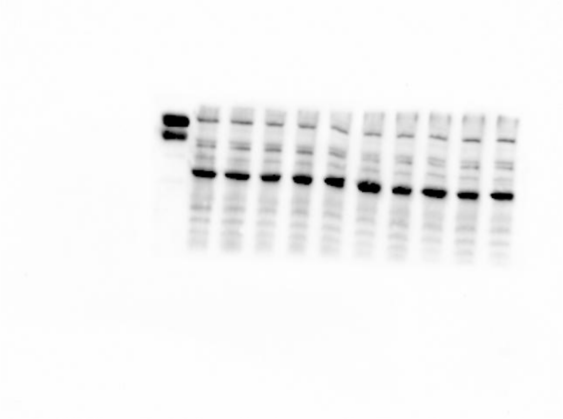

MKN-45P:

USP35

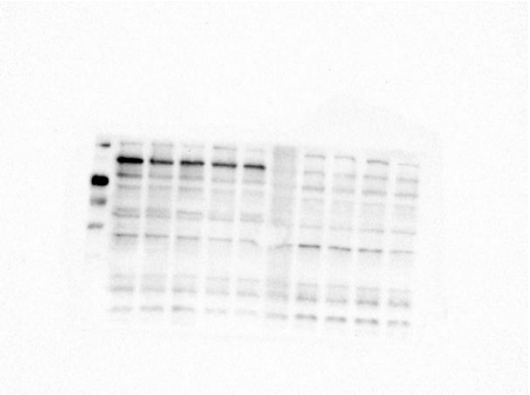

STING

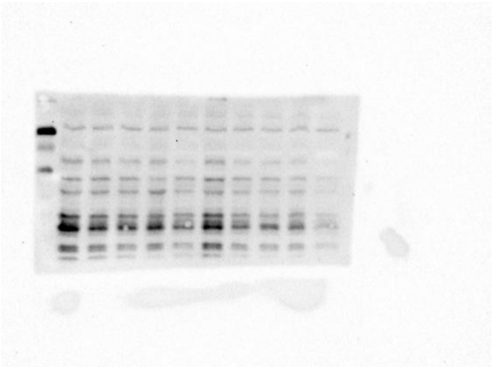

Actin

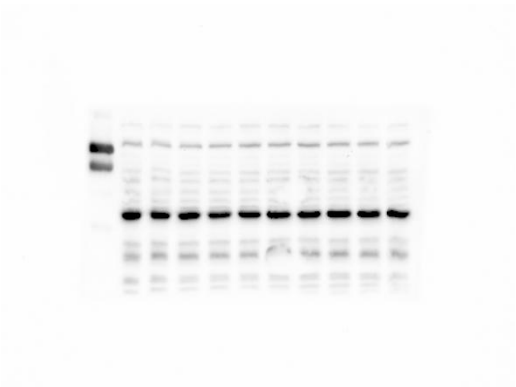

Figure 3D: MKN-45

STING

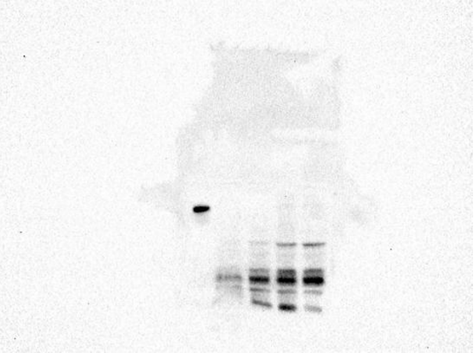

Actin

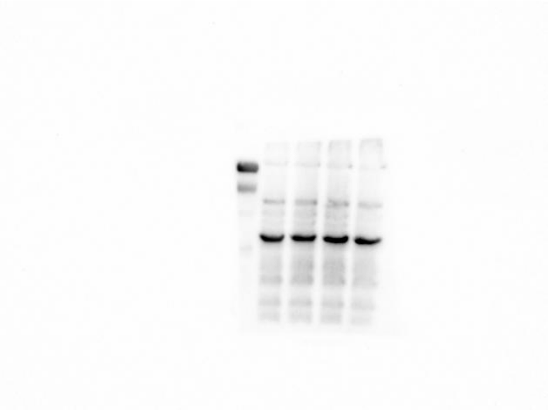

MKN-45P

STING

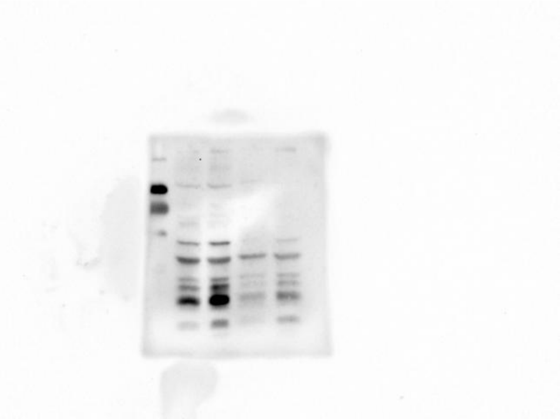

Actin

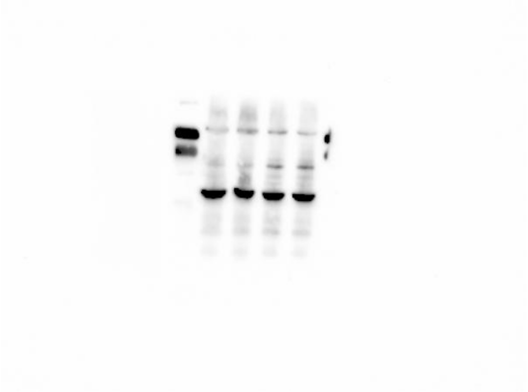

Figure 3E： STING IP USP35: MKN-45

USP35

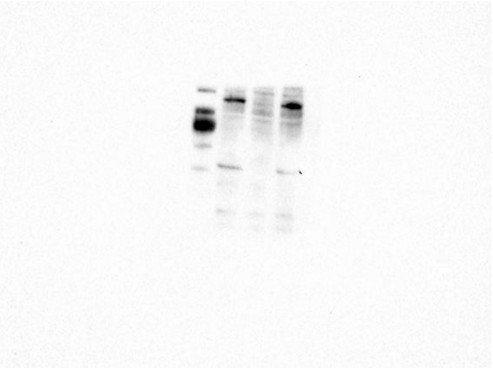

STING

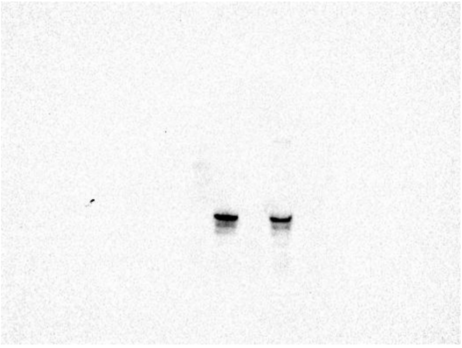

MKN-45P

USP35

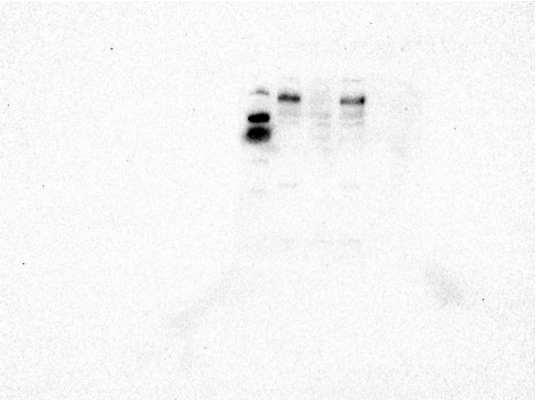

STING

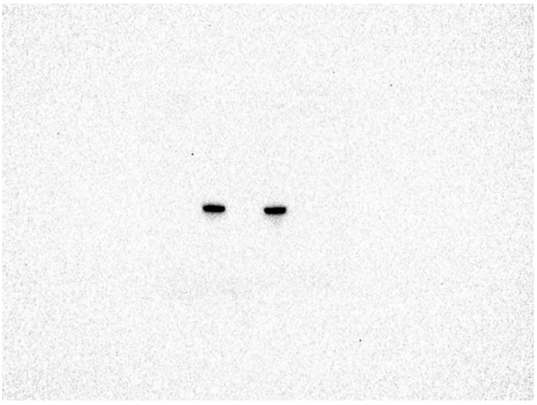

USP35 IP STING: MKN-45

USP35

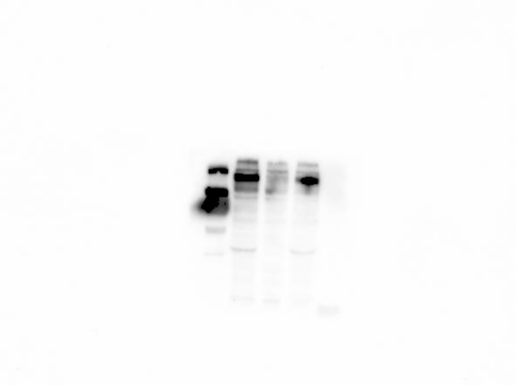

STING

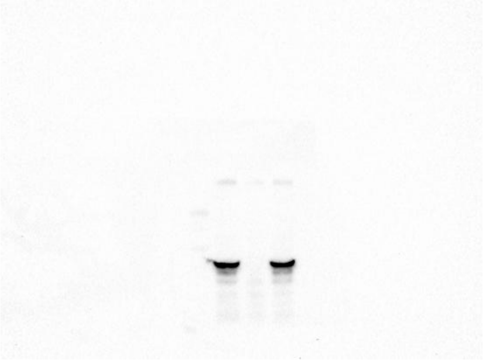

MKN-45P

USP35

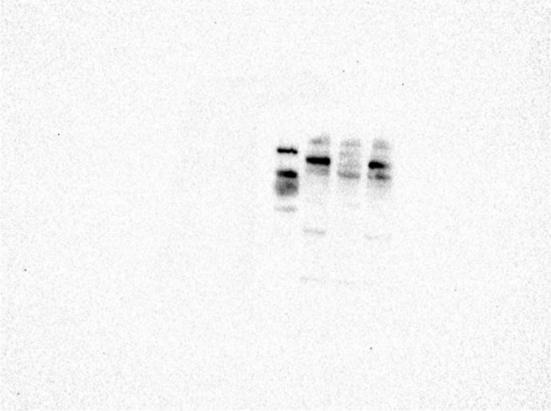

STING

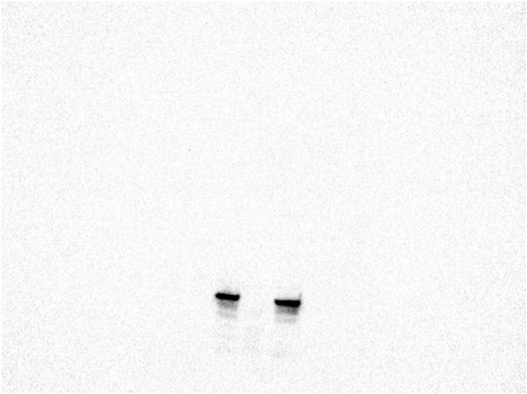

Figure 3F： MKN-45

Ub

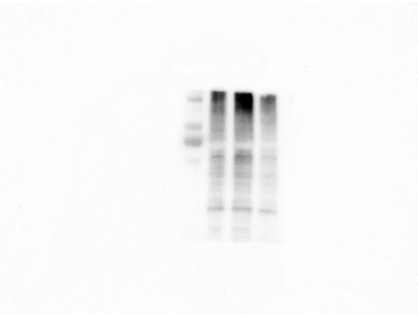

IP: USP35

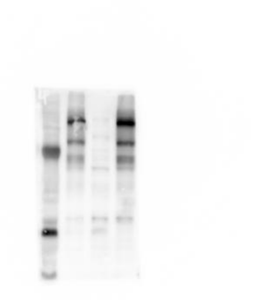

Actin

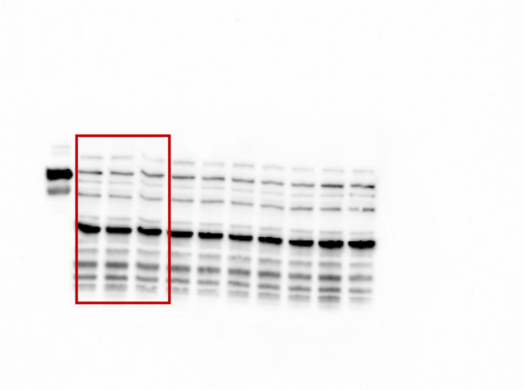

Input: USP35

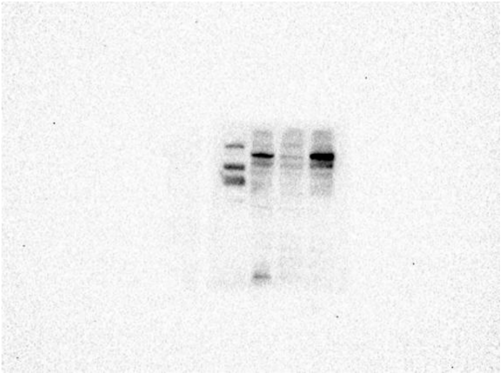

STING

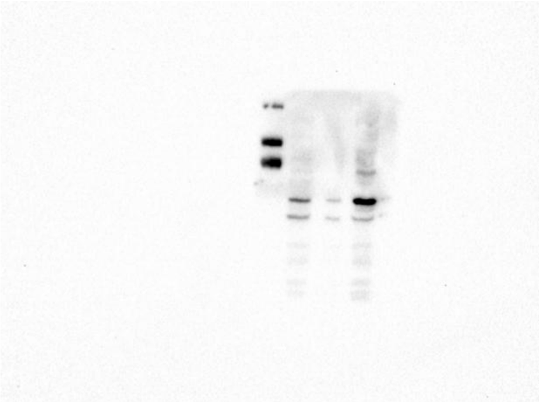

MKN-45P

Ub

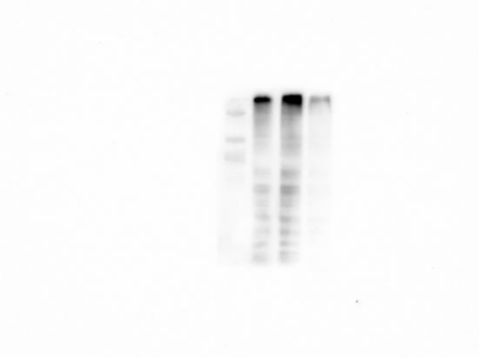

IP: USP35

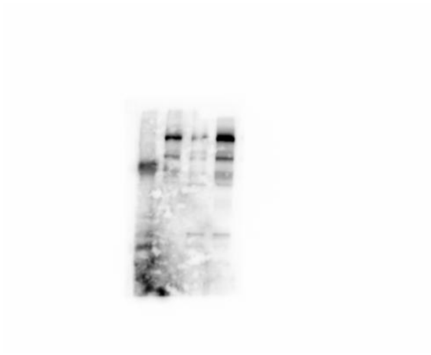

Actin

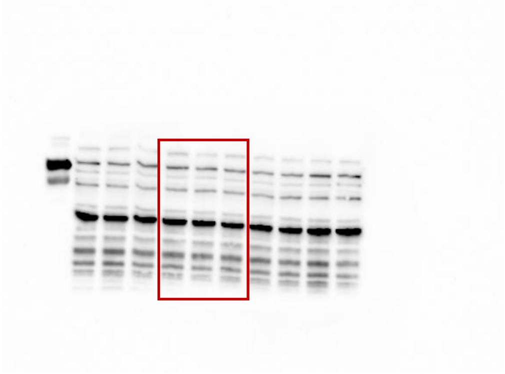

Input: USP35

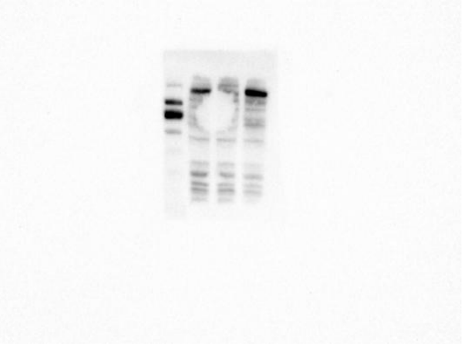

STING

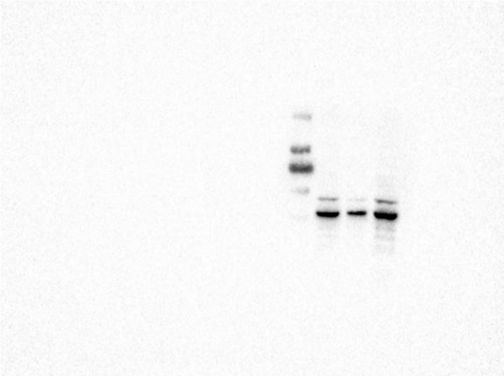

Figure 3G:

MKN-45

HA-ub

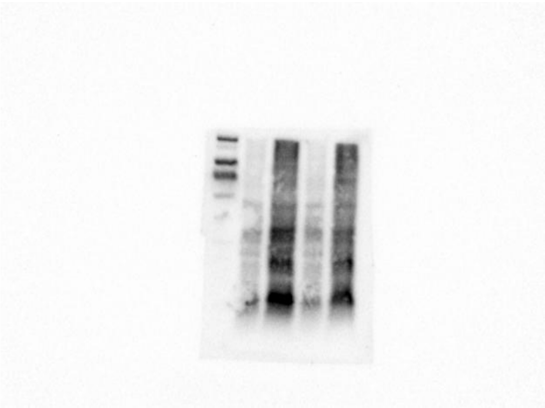

flag-STING IP

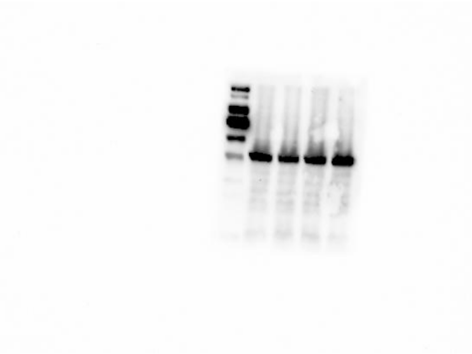

Flag-STING IB

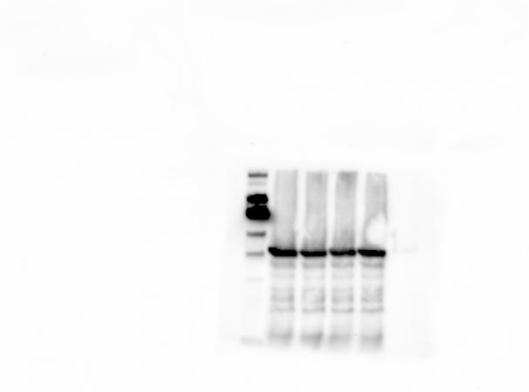

USP35

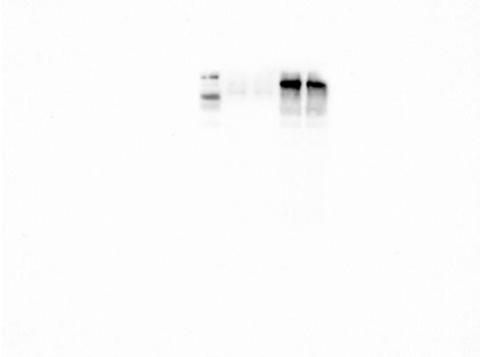

MKN-45P

HA-ub

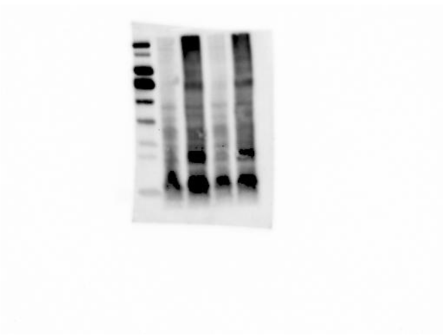

flag-STING IP

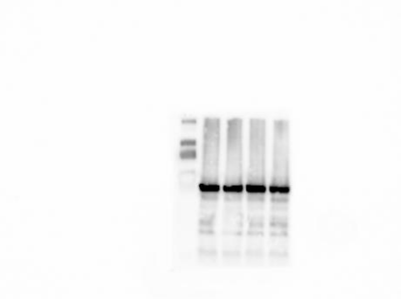

Flag-STING IB

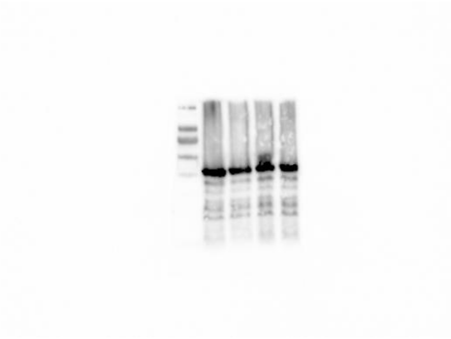

USP35

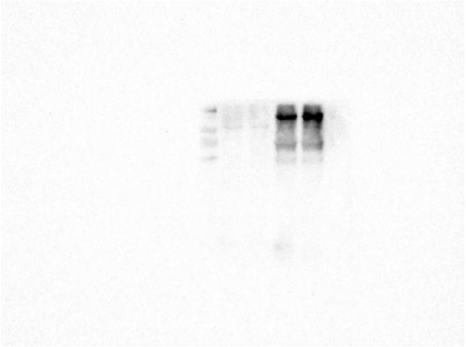

Figure 3H:

HA-ub WT K6 K11 K27

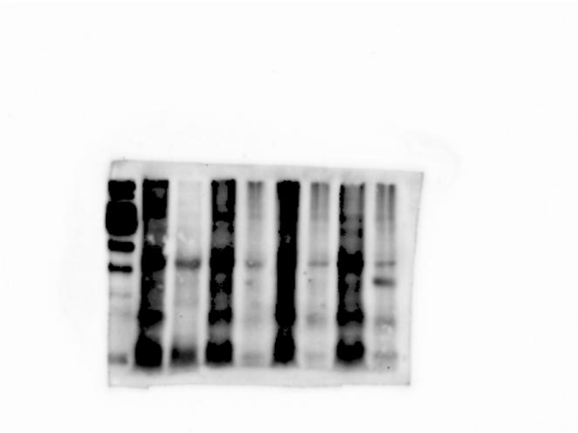

HA-ub K29 K33 K48 K63

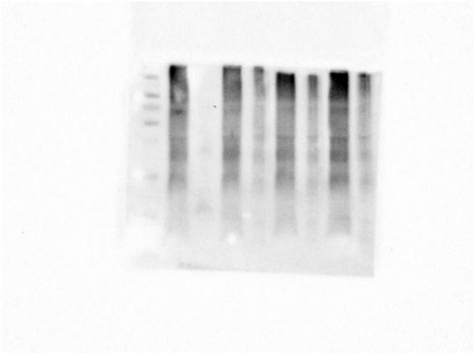

Flag-STING IP WT K6 K11 K27

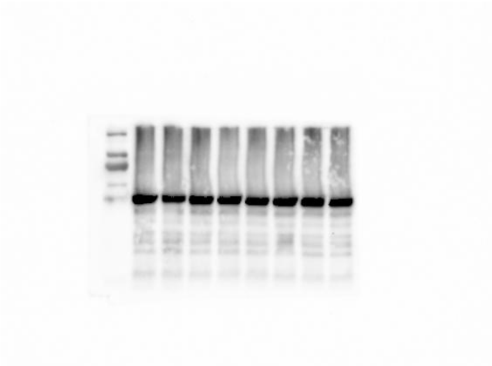

Flag-STING IP K29 K33 K48 K63

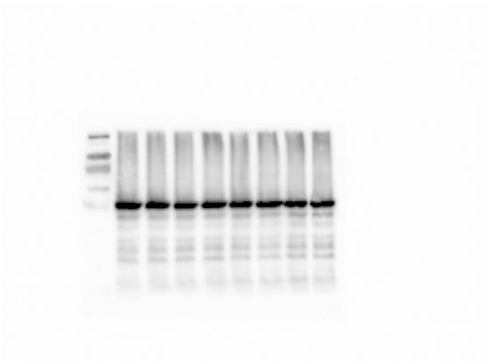

Flag-STING IB WT K6 K11 K27

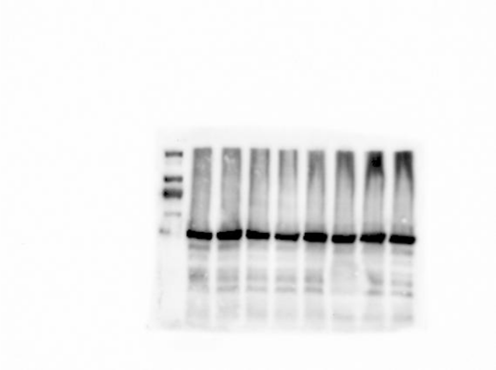

Flag-STING IB K29 K33 K48 K63

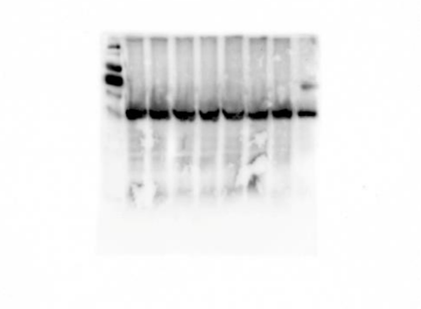

Myc-USP35 IB WT K6 K11 K27

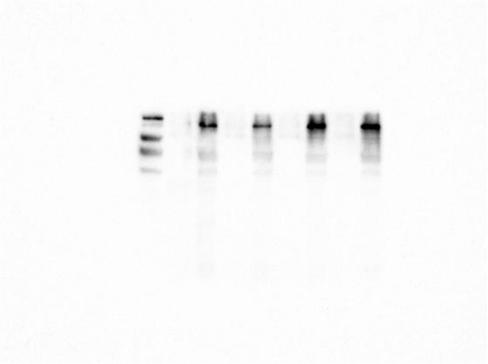

Myc-USP3 IB K29 K33 K48 K63

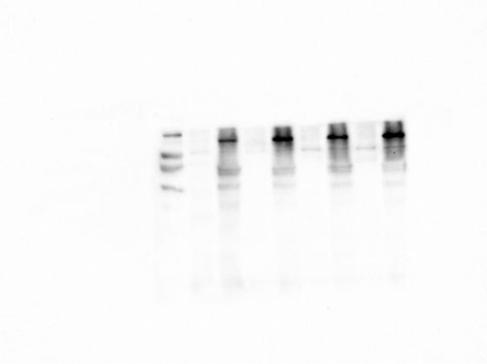

Figure 4E:

sh-USP35/OE-STING

USP35

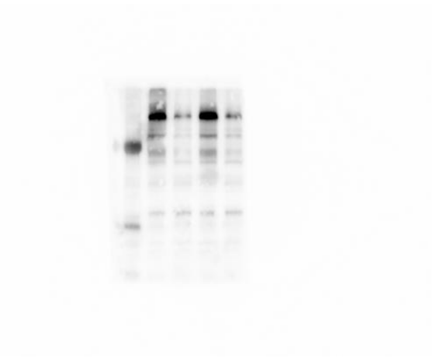

STING

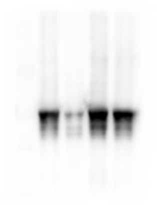

ICAM1

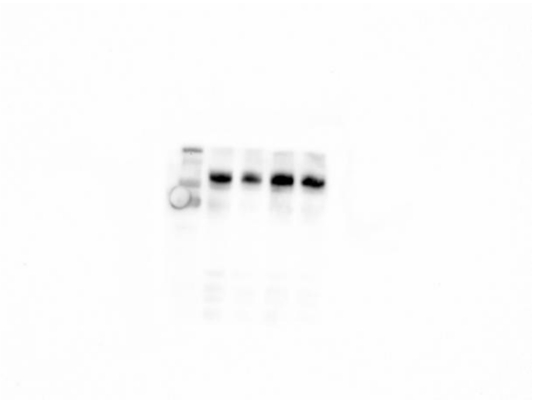

e-selectin

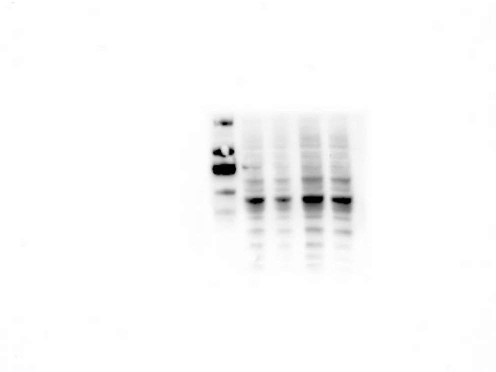

HIF-1A

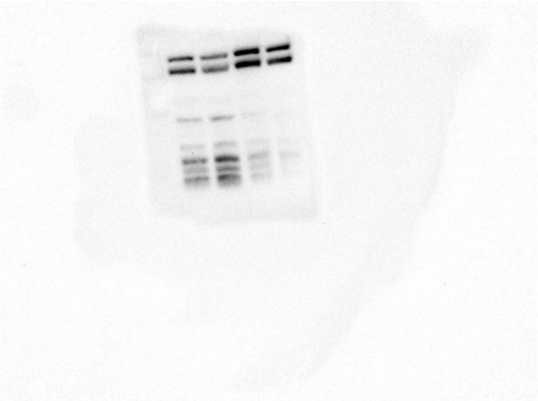

P-FAK

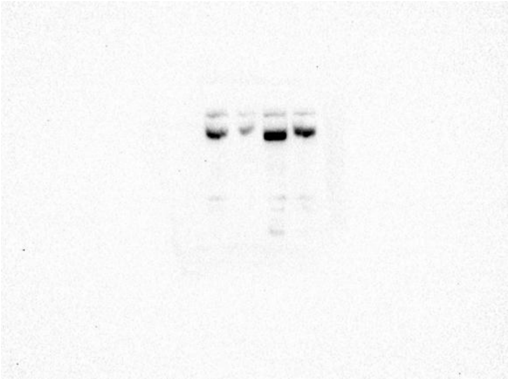

FAK

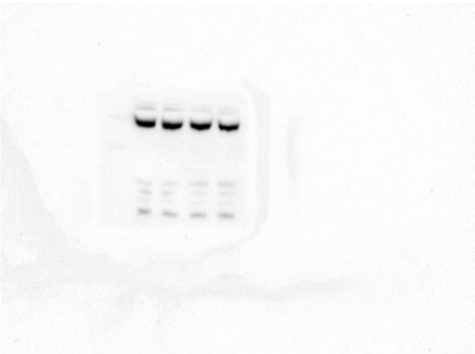

Actin

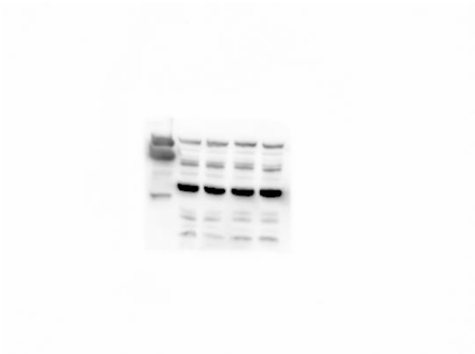

sh-USP35/C-176

USP35

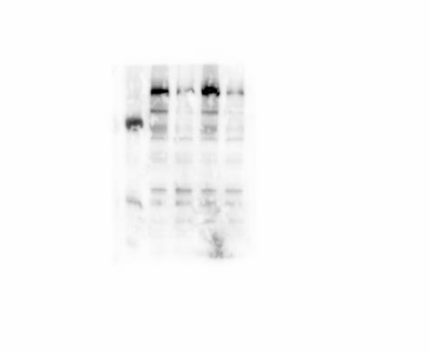

STING

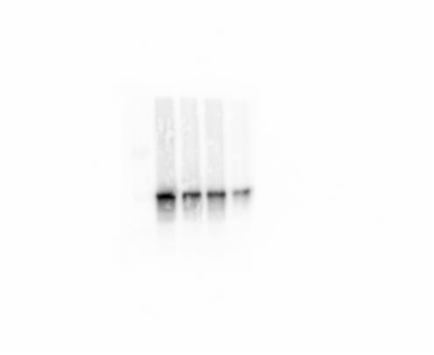

ICAM1

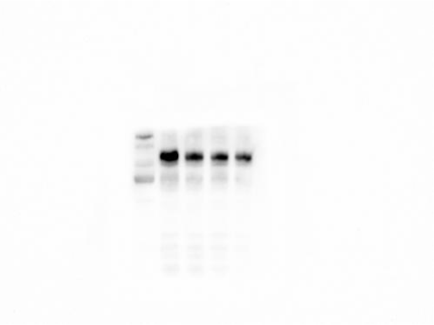

e-selectin

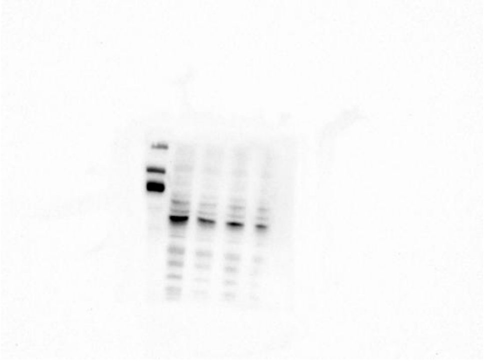

HIF-1A

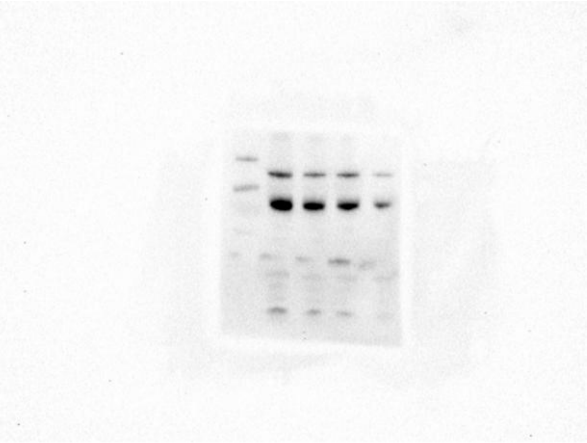

p-FAK

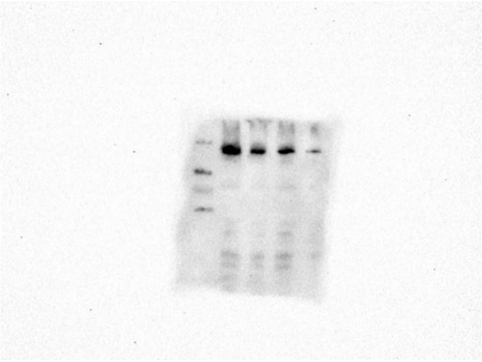

FAK

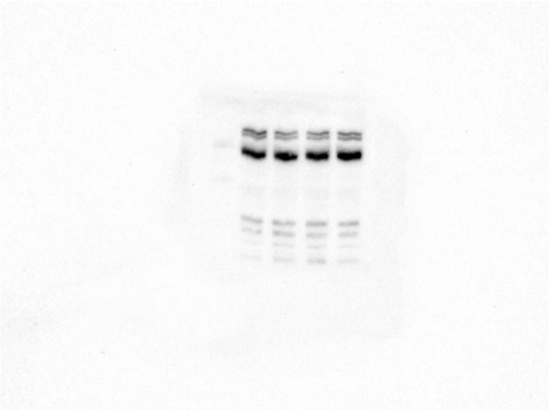

Actin

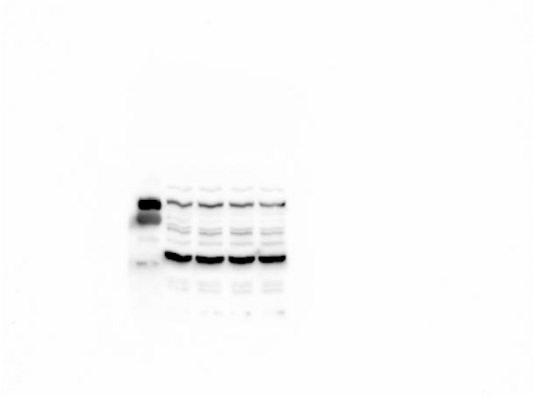

Figure 4G:

HIF-1A

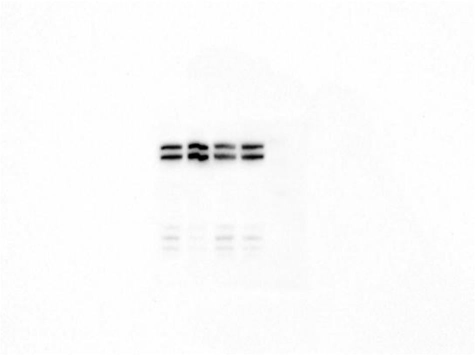

ICAM1

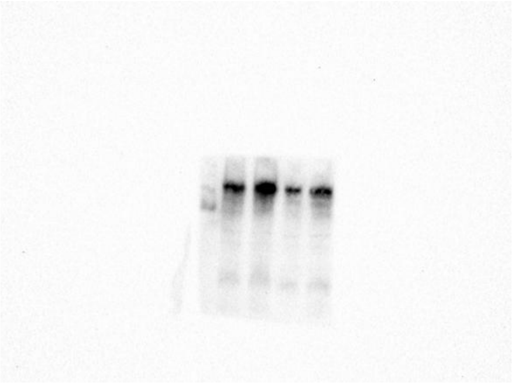

e-selectin

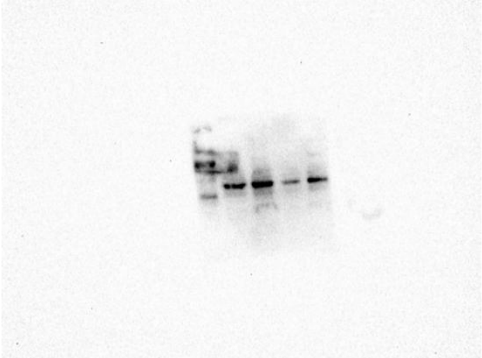

p-FAK

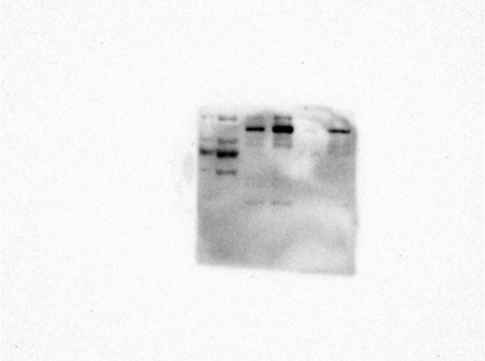

FAK

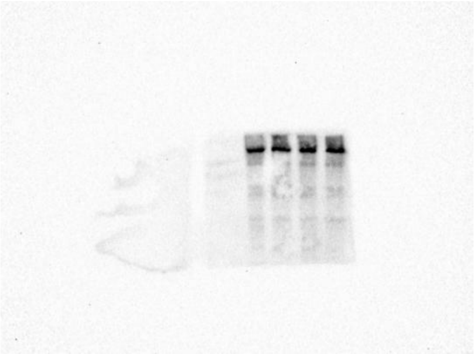

Actin

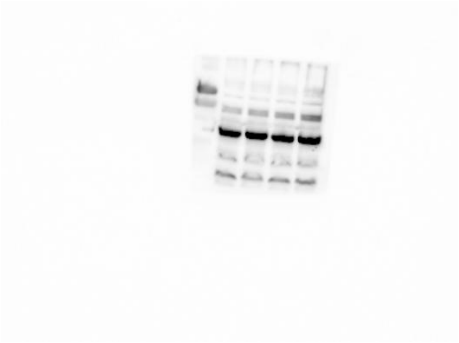

Figure 5B: GES-1

TSG101

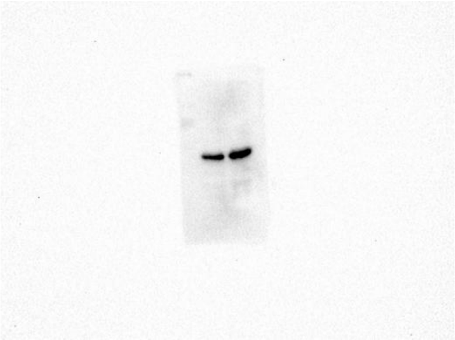

CD81

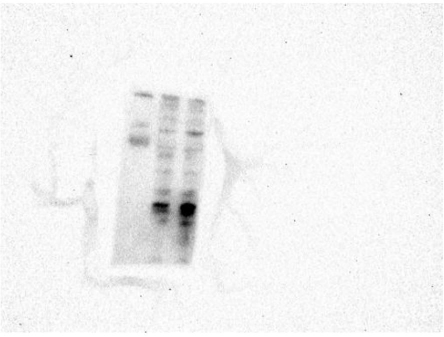

Calnexin

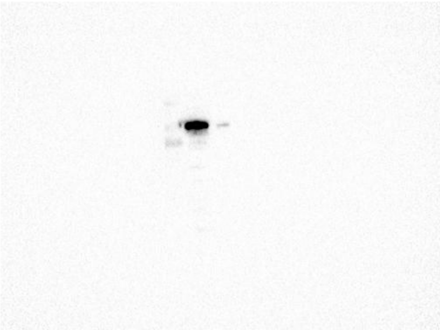

MKN-45

TSG101

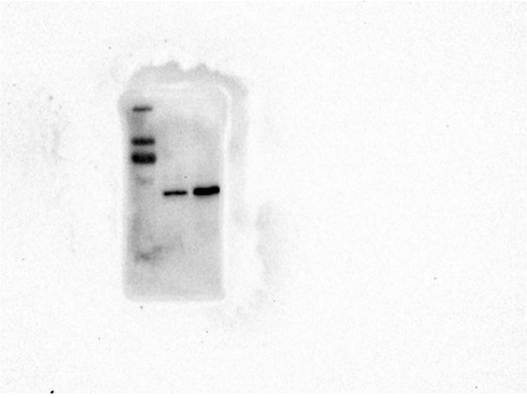

CD81

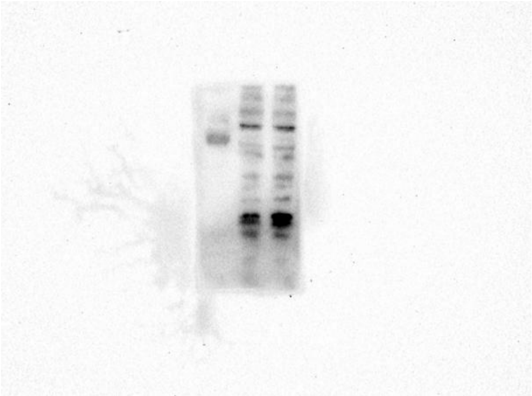

Calnexin

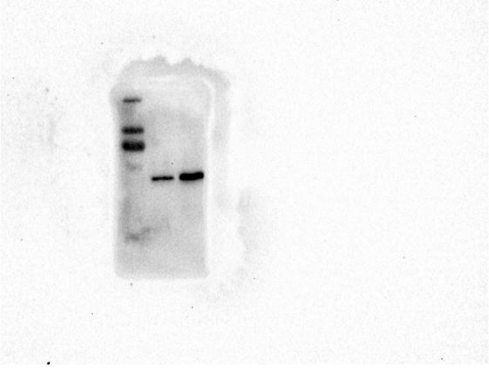

MKN-45P

TSG101

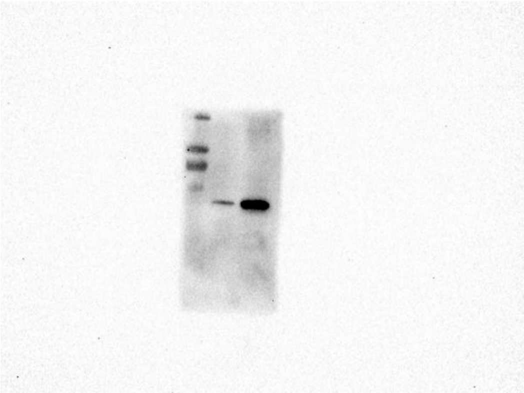

CD81

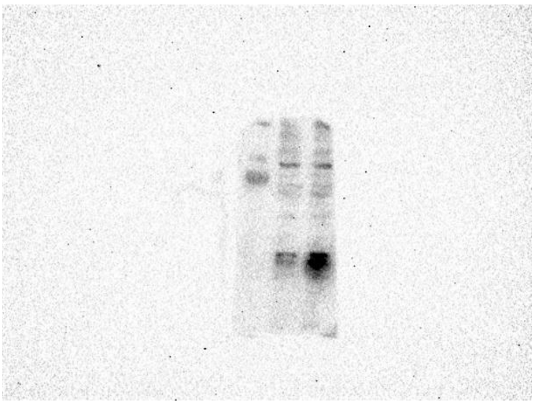

Calnexin

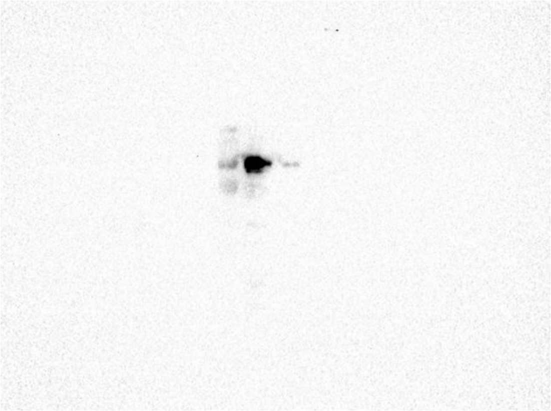

Figure 5D：

USP35

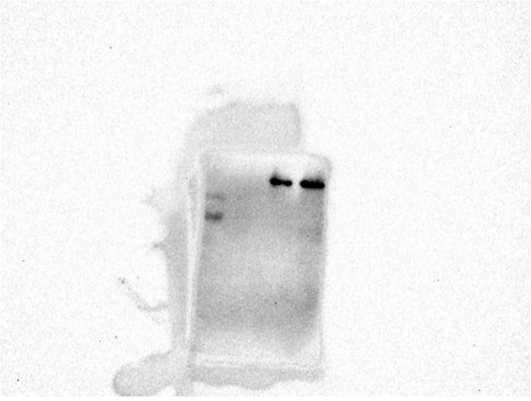

TSG101

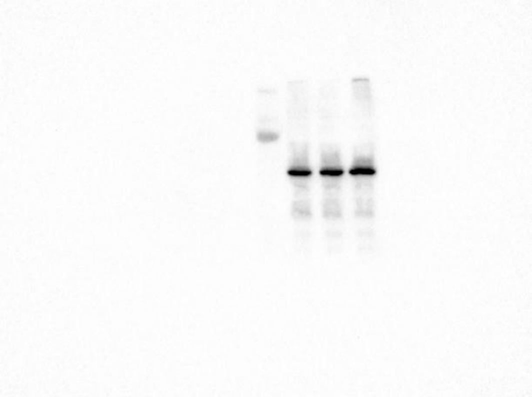

Figure 5F：MKN-45

TSG101

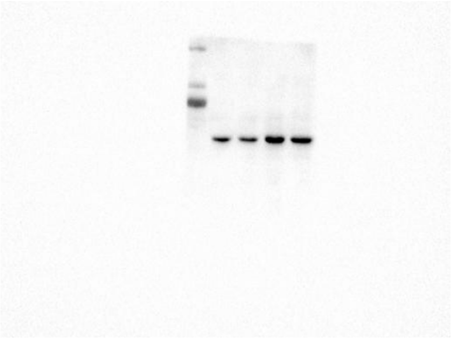

CD81

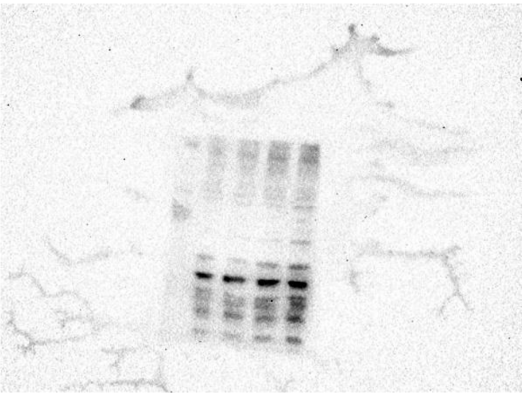

Calnexin

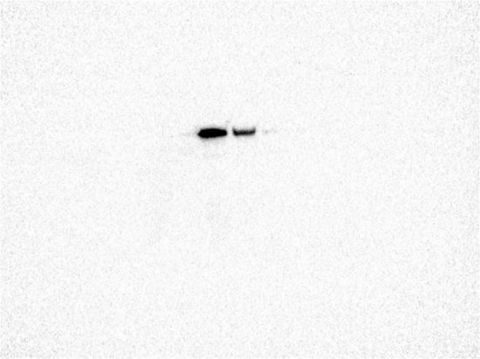

Figure 5F：MKN-45P

TSG101

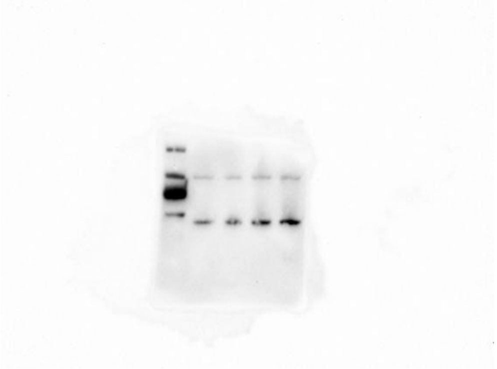

CD81

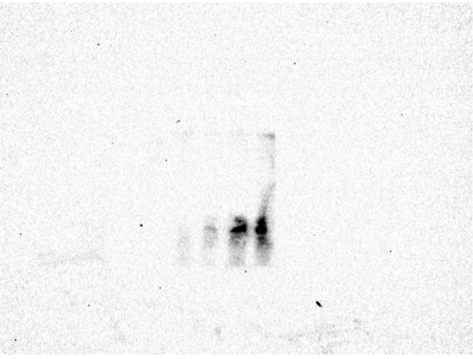

Calnexin

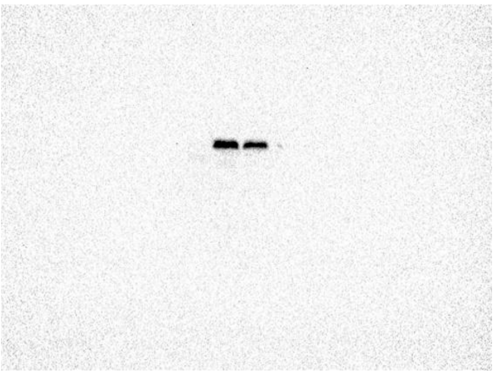

Figure 5G：MKN-45

USP35

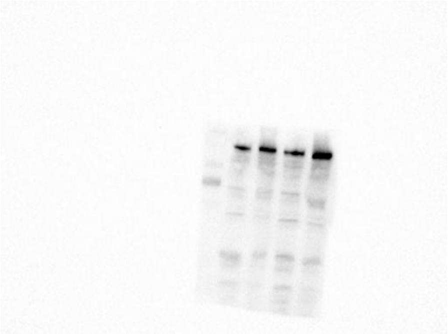

TSG101

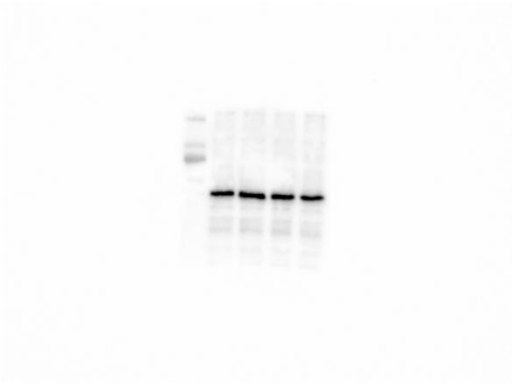

MKN-45P：

USP35

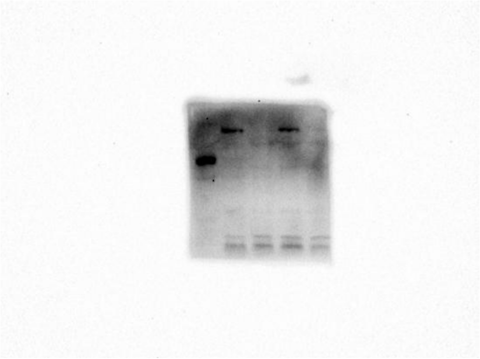

TSG101

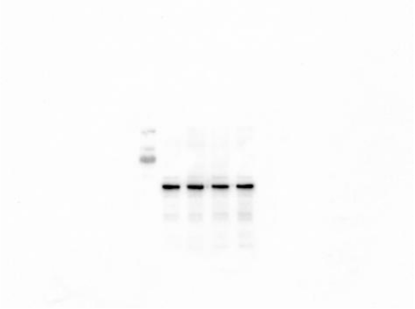

Figure 5I：MKN-45

E-cadherin

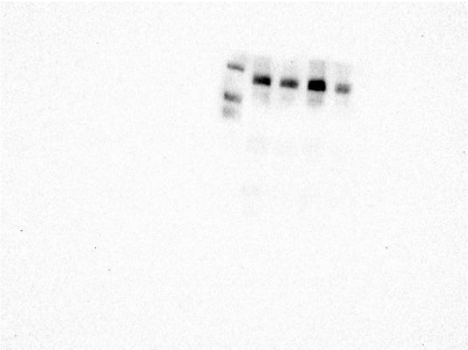

Fibronectin

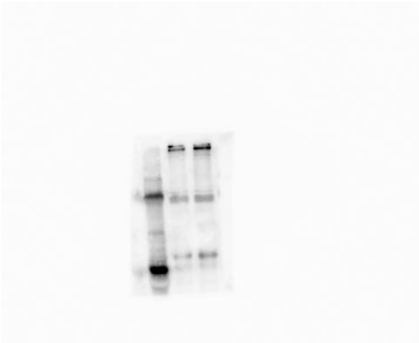

α-SMA

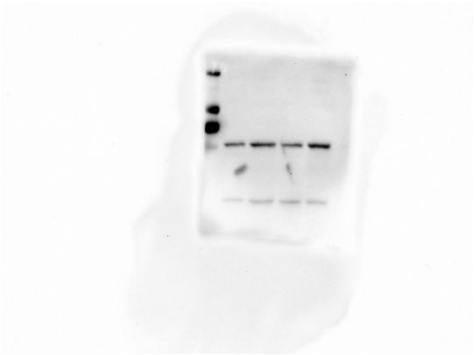

actin

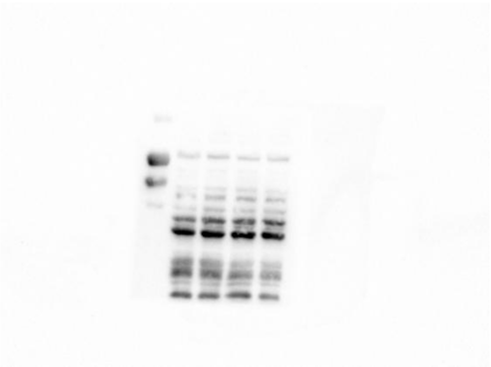

MKN-45P

E-cadherin

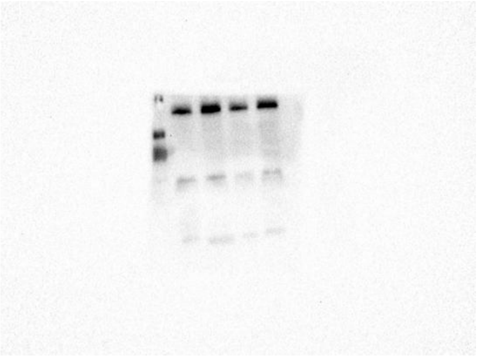

Fibronectin

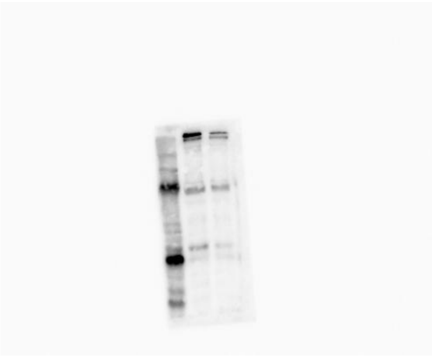

α-SMA

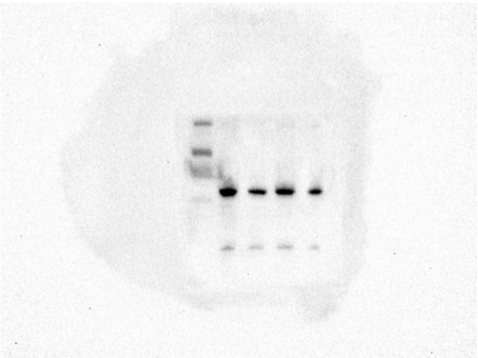

actin

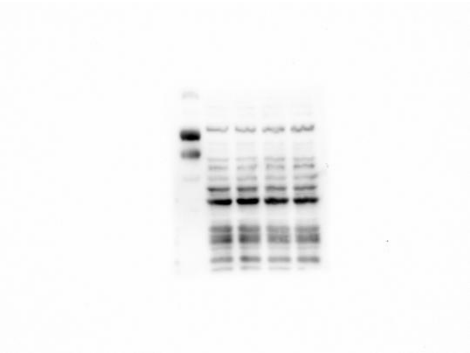

Figure S1B:

AGS/HGC-27:

USP35 actin

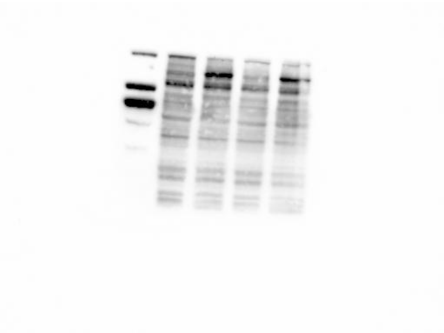

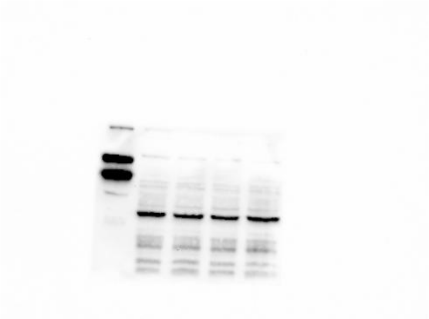

MKN-45:

USP35 actin

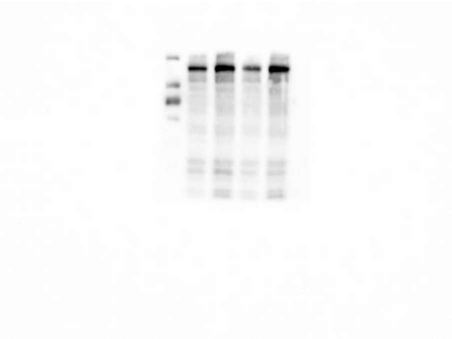

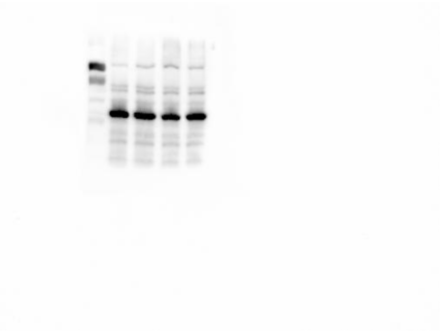

Figure S1D:

USP35 actin

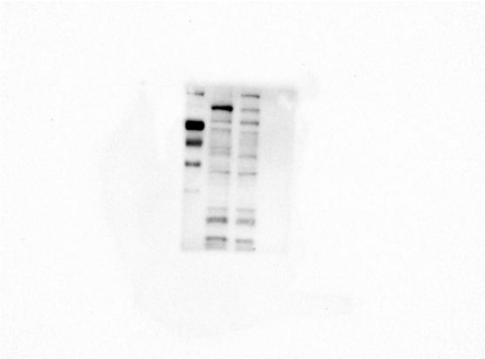

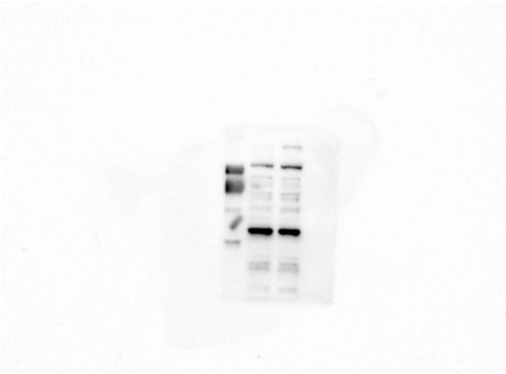

Figure S1F:

STING actin

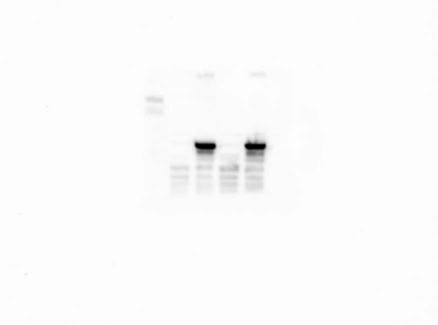

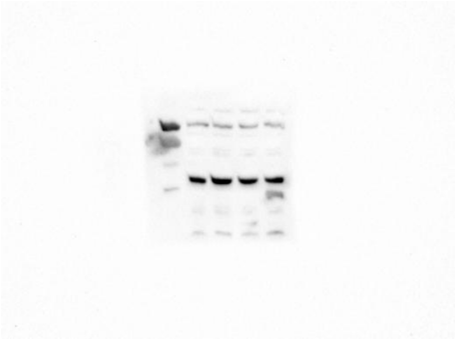

Figure S2E:

ICAM1

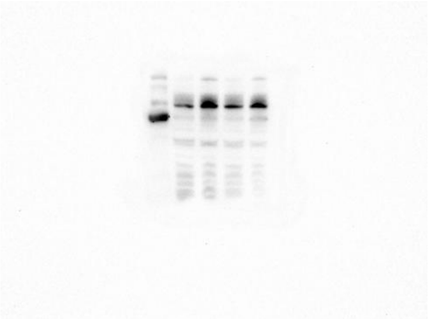

e-selectin

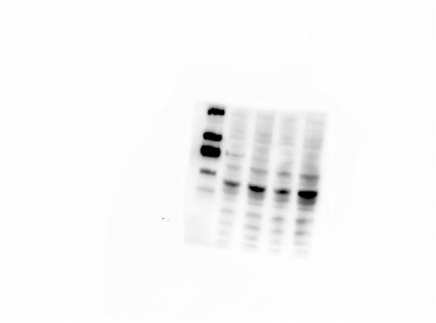

Actin

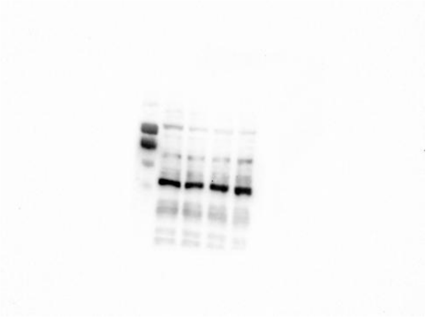

Supplement: Supplementary file 2 — Western blot [file 41419_2025_8322_MOESM2_ESM.pdf]
